# Collocation of Next-Generation Operators for Computing the Basic Reproduction Number of Structured Populations

**DOI:** 10.1007/s10915-020-01339-1

**Published:** 2020-10-31

**Authors:** Dimitri Breda, Toshikazu Kuniya, Jordi Ripoll, Rossana Vermiglio

**Affiliations:** 1 CDLab – Computational Dynamics Laboratory, Department of Mathematics, Computer Science and Physics, University of Udine, via delle scienze 206, 33100 Udine, Italy; 2 Graduate School of System Informatics, Kobe University, 1-1 Rokkodai-cho, Nada-ku, Kobe, 657-8501 Japan; 3 Department of Computer Science, Applied Mathematics and Statistics, University of Girona, Campus Montilivi, 17003 Girona, Spain

**Keywords:** Pseudospectral collocation, Spectral approximation, Spectral radius, Next-generation operator, Basic reproduction number, Stability analysis of equilibria, Structured population dynamics, 65J10, 65L03, 65L15, 65M70, 37N25, 47D06, 47A75, 92D25, 92D30, 92D40

## Abstract

We contribute a full analysis of theoretical and numerical aspects of the collocation approach recently proposed by some of the authors to compute the basic reproduction number of structured population dynamics as spectral radius of certain infinite-dimensional operators. On the one hand, we prove under mild regularity assumptions on the models coefficients that the concerned operators are compact, so that the problem can be properly recast as an eigenvalue problem thus allowing for numerical discretization. On the other hand, we prove through detailed and rigorous error and convergence analyses that the method performs the expected spectral accuracy. Several numerical tests validate the proposed analysis by highlighting diverse peculiarities of the investigated approach.

## Introduction

In the wide field of population dynamics, including both ecological and epidemic models, the *basic reproduction number* $R_{0}$ is a key quantity in tackling important evolutionary aspects, see, e.g., [3, 18] as starting references. In mathematical epidemiology, for instance, $R_{0}$ measures the average number of secondary cases produced by a typical infected individual in a fully susceptible population, thus indicating the intensity of an epidemic in its initial stage. Estimating $R_{0}$ is thus a primary target during serious emerging infectious diseases. In this respect, coronavirus disease 2019 (COVID-19) is a last testament of such a critical scenario [26, 27, 32]. As a second instance, within-host dynamics gives rise to rich ecological models which are underneath the infection processes. The computation of $R_{0}$ is also important for these type of models as long as the evolutionary standpoint is concerned [9].

A novel and efficient numerical method for computing $R_{0}$ for structured population dynamics is introduced in [5] by some of the authors. Therein, a thorough experimental investigation demonstrates both the applicability and the validity of the approach, thus revealing an effective tool in the analysis of a comprehensive range of ecological and epidemic models, based on either first and second order (ordinary and partial) differential operators, as well as integro-differential ones. The characterization of $R_{0}$ as the spectral radius of a certain positive operator, known as the *Next-Generation Operator* (NGO), is favorably exploited to turn the relevant infinite-dimensional eigenvalue problem into a finite-dimensional one. Dominant eigenvalues of the latter are then used as approximations to dominant eigenvalues of the former. The underlying discretization is based on pseudospectral collocation, suitably accounting for the boundary conditions defining the NGO.

The present work completes the theoretical and numerical analysis of the approach proposed in [5] by bringing two fundamental contributions. On the one hand, we prove that under reasonable and mild regularity assumptions on the coefficients of the models of interest the NGO is compact, so that $R_{0}$ is indeed a positive eigenvalue according to the celebrated result of Krein and Rutman [23]. On the other hand, most of the efforts are devoted to develop a complete and fully detailed error analysis, together with the relevant results about convergence. Concerning the latter, let us remark that the numerical discretization of one of the two classes of models considered in [5] (viz., model A) is slightly modified here in order to facilitate the convergence analysis, with no essential qualitative or quantitative difference in the numerical results. Moreover, it is worthy to mention that the error analysis relies on constructing exact and approximated characteristic equations and following the approach of [6] or [7, Chapter 5] as abstract guidelines.

Together with [5], the current research represents a framework of reference for the numerical computation of $R_{0}$ in both ecology and epidemiology. In this respect, the general outcome is a quite reliable tool, with faster convergence ideally of infinite order, a feature known as *spectral accuracy*, see, e.g., [37]. The latter is a great advantage compared to the finite-order convergence of the only two preceding methods [17, 25], based respectively on $\theta$- and Euler discretization schemes. This advantage translates into much more accurate approximations obtained with much smaller matrices, leading to a reduced computational load, in terms of both time and memory. This is a favorable feature when stability and bifurcation analyses are the final target in presence of varying or uncertain model parameters, as is frequently the case in realistic contexts. In this respect, to note the work [15] concerning the sensitivity analysis of the computation of $R_{0}$.

The contents are organized as follows. In Sect. 2 we resume from [5] the main ingredients of the two classes of models of interest, namely *model A* for ecology and *model B* for epidemics. Compactness of the relevant NGO is proved in Sect. 3. The numerical treatment is illustrated in Sect. 4, including discretization, error analysis and convergence for both classes. In Sect. 5 we validate the obtained convergence results with several experiments, enlarging the benchmark initiated in [5]. Concluding remarks and potential future extensions are considered in Sect. 6. Note that Sects. 4.1 and 4.2 contain explicit expressions of the discretizing matrices for the sake of implementation (Matlab demos are freely available at http://cdlab.uniud.it/software).

## Models and Theoretical Background

Let $l>0$ be real and finite, and *X* be a Banach lattice of functions $[0,l]\rightarrow {\mathbb {R}}$. In the sequel $u\in X$ represents the density of individuals of a population, structured by, e.g., age, size or space^1^.

We are interested in abstract linear evolution equations of the form

1 $$\begin{aligned} u'(t)=Bu(t)-Mu(t),\quad u(t)\in X,\;t\ge 0. \end{aligned}$$

$B:X\rightarrow X$ is a linear operator meant to account for a *birth* process, would it be either proper birth, as in ecological models, or infection, as in epidemics. $M:{\mathcal {D}}(M)\subseteq X\rightarrow X$ is a linear operator meant to account for all the other processes, which we call *mortality* for brevity, would it be either proper mortality, as in ecological models, or any stage transition, as in epidemics (e.g., recovery or quarantine, Part 2 of [35]). Note that, typically,

2 $$\begin{aligned} {\mathcal {D}}(M)=\{\phi \in Y\ :\ {\mathcal {C}}\phi =0\} \end{aligned}$$

for $Y\subseteq X$ a subspace characterized by some degree of smoothness and additional constraints expressed through the linear map ${\mathcal {C}}:X\rightarrow {\mathbb {R}}^{p}$ for some positive integer *p*. From the modelling point of view, one should be aware that the classification of the terms in the right-hand side of (1) into either *B* or *M* is not unique (see, e.g., [2]). Finally, the following assumptions are as common as biologically meaningful (see, e.g., [3, 30, 34]):

1. *B* is positive and bounded;
2. $-M$ generates a strongly-continuous semigroup $\{T(t)\}_{t\ge 0}$ of positive linear operators, with strictly negative spectral bound.

Note that (A2) guarantees extinction in absence of birth, as well as the invertibility of *M* with $M^{-1}=\int _{0}^\infty T(t)\mathrm{d}t$.

The class of equations (1) represents a family of population models for which we can define the NGO as $BM^{-1}:X\rightarrow X$. This family is indeed rather large, see, e.g., [11, Section 7.2] and [3]. Then we can characterize the basic reproduction number as the spectral radius of the NGO, i.e.,

3 $$\begin{aligned} R_{0}:=\rho (BM^{-1}). \end{aligned}$$

The theoretical framework for the basic reproduction number is well-established, see, e.g., [11, 12, 18, 21] and the references therein. Under (A1) and (A2) $BM^{-1}$ is positive and bounded^2^, so that $R_{0}$ is a non-negative spectral value, see, e.g., [34]. If, in addition, $BM^{-1}$ is also compact and it has positive spectral radius, then the Krein-Rutman theorem^3^[23] ensures that $R_{0}$ is a positive eigenvalue, i.e., a solution $\lambda >0$ of

4 $$\begin{aligned} BM^{-1}\psi =\lambda \psi \end{aligned}$$

for some (nontrivial) positive eigenfunction $\psi$. Equivalently, $\lambda$ satisfies the *generalized* eigenvalue problem

5 $$\begin{aligned} B\phi =\lambda M\phi \end{aligned}$$

with $$\phi =M^{-1}\psi \in {\mathcal {D}}(M)$$.

Let us remark that the compactness of the NGO is a working hypothesis in the current study. Indeed, the numerical method under investigation relies on this assumption, in that (5) can be reduced to a standard (read finite-dimensional) generalized eigenvalue problem for matrices, whose dominant eigenvalue is thus used to approximate $R_{0}$. Concerning this numerical computation, a discussion on the relevance of $R_{0}$ being a generalized eigenvalue or not is left to Sect. 5. Here we first summarize from [5] the features of the two prototype classes of models we are interested in, taken respectively from ecology (in the sequel briefly *model A*) and epidemiology (*model B*). Then, in Sect. 3, we prove compactness for both classes under mild assumptions.

Eventually, the sign relation ${{\,\mathrm{sgn}\,}}(R_{0}-1)={{\,\mathrm{sgn}\,}}(r)$ is well-known, where *r* is the Malthusian parameter, i.e., the spectral bound of the complete generator $B-M$ (see, e.g., [36]). Although both approaches are equivalent for the analysis of population dynamics, the one based on $R_{0}$ has some advantages over the one based on *r* from both the theoretical and the numerical viewpoints (see further comments in [5]).

### Model A: Spatially-Structured Cell Populations

As a prototype representative model we consider a population of bacteria proliferating and moving along the intestine of an animal host, with no flux at the boundaries, see, e.g., [2, 3, 19]. As we are interested in the stability of the extinction equilibrium, we focus on the linear(ized) problem

6 $$\begin{aligned} \left\{ \begin{array}{ll} \partial _{t} u(x,t)+\partial _{x}\left[ c(x)u(x,t)-D(x)\partial _{x} u(x,t)\right] &{}\\ \qquad +[\beta (x)+\mu (x)]u(x,t)=2\beta (x)u(x,t),&{}\quad x\in [0,l],\;t\ge 0,\\ c(\bar{x})u(\bar{x},t)-D(\bar{x})\partial _{x}u(\bar{x},t)=0,&{} \quad \bar{x}\in \{0,l\},\;t\ge 0, \end{array}\right. \end{aligned}$$

where $u(\cdot ,t)\in X:=L^{1}([0,l],{\mathbb {R}})$ is the spatial density of bacteria at time $t\ge 0$ along the intestine, the latter portrayed as the line segment [0, *l*]. Above, *c* is the velocity of the flow, *D* is the diffusion coefficient, $\beta$ is the fertility rate and $\mu$ is the mortality rate. Moreover, in (6), symmetric division is assumed without loss of generality (when a mother cell divides then two daughter cells are born and the former disappears). Further details about model A can be found in [5].

In order for (1) to describe (6), let us define the birth operator $B:X\rightarrow X$ as

7 $$\begin{aligned} (B\phi )(x):=2\beta (x)\phi (x),\quad x\in [0,l], \end{aligned}$$

and the mortality operator $M:{\mathcal {D}}(M)\subseteq X\rightarrow X$ as

8 $$\begin{aligned} (M\phi )(x):=\left[ c(x)\phi (x)-D(x)\phi '(x)\right] '+[\beta (x)+\mu (x)] \phi (x),\quad x\in [0,l], \end{aligned}$$

with domain

9 $$\begin{aligned} {\mathcal {D}}(M)&:=\big \{\phi \in X\ :\ \phi ',(c\phi -D\phi ')'\in X\text { and }\nonumber \\&\qquad c(\bar{x})\phi (\bar{x})-D(\bar{x})\phi '(\bar{x})=0\text { for } \bar{x} \in \{0,l\}\big \}. \end{aligned}$$

Finally, for the sake of readability, we collect here a list of hypotheses concerning the model coefficients that are used at possibly different points later in the text (hereafter the subscript $+$ denotes the positive cone of the relevant Banach lattice):

1. $c,D,\beta ,\mu \in X_{+}$ with $\beta +\mu$ not identically vanishing;
2. $$\beta \in L^{\infty }_{+}([0,l],{\mathbb {R}})$$;
3. $$D(x)\ge \tilde{D}>0$$ for any $$x\in [0,l]$$;
4. $$c,D,\beta ,\mu \in X_{+}$$ are continuous;
5. $c,D,\beta ,\mu \in X_{+}$ are smooth, in particular
  (HA5.1) $c,D,\beta ,\mu \in X_{+}$ are of class $C^{s}$ for some integer $s\ge 1$;
  (HA5.2) $c,D,\beta ,\mu \in X_{+}$ are of class $C^{\infty }$;
  (HA5.3) $c,D,\beta ,\mu \in X_{+}$ are real analytic.

### Model B: Age-Structured Epidemics

As a prototype representative model we consider the spread of an infectious disease in an age-structured population. As we are interested in the early stage of the epidemics, we focus on the linear(ized) problem

10 $$\begin{aligned} \left\{ \begin{array}{ll} &{}\displaystyle \partial _{t} u(x,t)+\partial _{x}u(x,t) =\int _{0}^{l}K(x,y)u(y,t)\mathrm{d}y \\ &{}\quad -\eta (x)u(x,t), \quad x\in [0,l],\;t\ge 0,\\ &{}\displaystyle u(0,t)=\theta \int _{0}^{l}\beta (x)u(x,t)\mathrm{d}x, \quad t\ge 0, \end{array}\right. \end{aligned}$$

where $u(\cdot ,t)\in X:=L^{1}([0,l],{\mathbb {R}})$ is the age density of infected individuals^4^ at time $t\ge 0$, *l* being the (finite chronological) maximum age. Above, *K* is the effective infection kernel, $\beta$ the effective fertility rate and $\eta$ accounts for both removal and recovery rates. Moreover, in (10), $\theta \in [0,1]$ is the the probability of vertical transmission of infectiveness. Further details about model B can be found in [5].

In order for (1) to describe (10), let us define the birth^5^ operator $B:X\rightarrow X$ as

11 $$\begin{aligned} (B\phi )(x):=\int _{0}^{l}K(x,y)\phi (y)\mathrm{d}y,\quad x\in [0,l], \end{aligned}$$

and the mortality^6^ operator $M:{\mathcal {D}}(M)\subseteq X\rightarrow X$ as

12 $$\begin{aligned} (M\phi )(x):=\phi '(x)+\eta (x)\phi (x),\quad x\in [0,l], \end{aligned}$$

with domain

13 $$\begin{aligned} {\mathcal {D}}(M):=\left\{ \phi \in X\ :\ \phi '\in X \text { and }\phi (0)=\theta \int _{0}^{l}\beta (x)\phi (x)\mathrm{d}x\right\} . \end{aligned}$$

Again, for the sake of readability, we collect here a list of hypotheses concerning the model coefficients that are used at possibly different points later in the text:

1. $$K\in L^{1}_{+}([0,l]^{2},{\mathbb {R}})$$ and $$\beta ,\eta \in X_{+}$$ with
  14 $$\begin{aligned} \int _{0}^{l}\beta (x)\mathrm{d}x=1; \end{aligned}$$
2. there exists $\bar{K}\in X_{+}$ such that $K(x,y)\le \bar{K}(x)$ holds for almost all $(x,y)\in [0,l]^{2}$ uniformly with respect to *y*;
3. $$\beta \in L^{\infty }_{+}([0,l],{\mathbb {R}})$$;
4. $\eta$ is continuous, as well as the map $x\mapsto K(x,y)$ uniformly with respect to almost all $y\in [0,l]$;
5. $\eta$ is smooth, as well as the map $x\mapsto K(x,y)$ uniformly with respect to almost all $y\in [0,l]$, in particular
  (HB5.1) $\eta$ is of class $C^{s}$ for some integer $s\ge 1$, as well as the map $x\mapsto K(x,y)$ uniformly with respect to almost all $y\in [0,l]$;
  (HB5.2) $\eta$ is of class $C^{\infty }$, as well as the map $x\mapsto K(x,y)$ uniformly with respect to almost all $y\in [0,l]$;
  (HB5.3) $\eta$ is real analytic, as well as the map $x\mapsto K(x,y)$ uniformly with respect to almost all $y\in [0,l]$;
6. $\beta$ is smooth, in particular
  (HB6.1) $\beta$ is of class $C^{s}$ for some integer $s\ge 1$;
  (HB6.2) $\beta$ is of class $C^{\infty }$;
  (HB6.3) $\beta$ is real analytic.

## Compactness of Next-Generation Operators

As already remarked after (5), the numerical approach we study in Sect. 4 is based on the assumption that the basic reproduction number $R_{0}$ is a generalized eigenvalue, i.e., a solution $\lambda$ of (5) for some (nontrivial) eigenfunction $\phi$. Compactness of the relevant NGO $BM^{-1}$ is a standard requirement guaranteeing the above property, together with the fact that this eigenvalue is real and positive thanks to the Krein-Rutman theorem [23]. Below we prove compactness for both models A and B. In this respect, note that under (A1) it is enough to show that $M^{-1}$ is compact. Therefore, we adopt also conditions on the model coefficients guaranteeing (A1).

### Proposition 1

(**compactness for model A**) Under (HA1), (HA2) and (HA3), the NGO $BM^{-1}$ defined through (7) and (8) with (9) is compact.

### Proof

(HA2) guarantees that *B* in (7) is bounded. Given $\psi \in X$, let us compute $\phi =M^{-1}\psi \in {\mathcal {D}}(M)$ to show that $M^{-1}$ is compact. By recalling (8) and by defining

15 $$\begin{aligned} \xi :=c\phi -D\phi ', \end{aligned}$$

we end up with the Initial Value Problem (IVP)

16 $$\begin{aligned} \left\{ \begin{array}{l} \begin{pmatrix} \phi '(x)\\ \xi '(x) \end{pmatrix} =A^{}(x) \begin{pmatrix} \phi (x)\\ \xi (x) \end{pmatrix} +\begin{pmatrix} 0\\ \psi (x) \end{pmatrix} ,\quad x\in [0,l],\\ \begin{pmatrix} \phi (0)\\ \xi (0) \end{pmatrix} = \begin{pmatrix} \alpha \\ 0 \end{pmatrix}, \end{array}\right. \end{aligned}$$

where

$$\begin{aligned} A^{}:=\begin{pmatrix} \displaystyle \frac{c}{D}&{}\displaystyle -\frac{1}{D}\\ \displaystyle -\beta -\mu &{}0 \end{pmatrix}. \end{aligned}$$

Indeed, from (9), $\xi (0)=0$ is used explicitly, while $\xi (l)=0$ is used implicitly to assign $\phi (0)=\alpha$ for some $\alpha >0$, see (17) below.

Now, for any $y\in [0,l]$, let *T*(*x*, *y*) for $x\in [0,l]$, $x\ge y$, be the principal matrix solution at *y* of the homogeneous part of (16), i.e., the matrix solution of

$$\begin{aligned} \left\{ \begin{array}{l} T'(x,y)=A(x)T(x,y),\quad x\in [0,l],\\ T(y,y)=I_{2}. \end{array}\right. \end{aligned}$$

Under (HA1) and (HA3), the IVP is posed in $L^{1}$, so $T'$ is an $L^{1}$-map and *T* is absolutely (hence uniformly) continuous. Moreover, the variation of constants formula gives

$$\begin{aligned} \begin{pmatrix} \phi (x)\\ \xi (x) \end{pmatrix} =T(x,0) \begin{pmatrix} \alpha \\ 0 \end{pmatrix} +\int _{0}^{x}T(x,y) \begin{pmatrix} 0\\ \psi (y) \end{pmatrix} \mathrm{d}y. \end{aligned}$$

In particular, we have

$$\begin{aligned} 0=\xi (l)=T_{2,1}(l,0)\alpha +\int _{0}^{l}T_{2,2}(l,y)\psi (y)\mathrm{d}y \end{aligned}$$

and

$$\begin{aligned} T_{2,1}(x,0)=-\int _{0}^{x}[\beta (y)+\mu (y)]T_{1,1}(y,0)\mathrm{d}y. \end{aligned}$$

If $T_{2,1}(l,0)=0$, then also $T_{2,2}(l,y)\equiv 0$ (since $\psi$ is arbitrary) and $T_{1,1}(y,0)\equiv 0$ (thanks to (HA1)), leading to *T*(*l*, 0) being singular, which is absurd. Hence we recover

17 $$\begin{aligned} \alpha =H(\psi ):=-(T_{2,1}(l,0))^{-1}\int _{0}^{l}T_{2,2}(l,y)\psi (y)\mathrm{d}y \end{aligned}$$

and, eventually,

18 $$\begin{aligned} (M^{-1}\psi )(x)= T_{1,1}(x,0)H(\psi )+\int _{0}^{x}T_{1,2}(x,y)\psi (y)\mathrm{d}y. \end{aligned}$$

To prove compactness, we resort to the Kolmogorov-Riesz-Fréchet theorem (see, e.g., Theorem 4.26 in [8]), and in this respect we extend all functions by zero outside [0, *l*]. Then fix $m>0$ and consider the set $U:=\{\psi \in X\ :\ \Vert \psi \Vert _{X}\le m\}$. It suffices to prove that

$$\begin{aligned} \lim _{h\rightarrow 0}\int _{{\mathbb {R}}}[(M^{-1}\psi )(x+h)-(M^{-1}\psi )(x)]\mathrm{d}x=0 \end{aligned}$$

uniformly with respect to $\psi \in U$. (18) gives

$$\begin{aligned}&\displaystyle \int _{{\mathbb {R}}}|(M^{-1}\psi )(x+h) -(M^{-1}\psi )(x)|\mathrm{d}x\le \displaystyle \int _{{\mathbb {R}}}| T_{1,1}(x+h,0)-T_{1,1}(x,0)|\mathrm{d}x\cdot |H(\psi )|\\&\qquad +\displaystyle \int _{{\mathbb {R}}}\int _{0}^{l}| T_{1,2}(x+h,y)-T_{1,2}(x,y)|\cdot |\psi (y)|\mathrm{d}y\mathrm{d}x\\&\qquad +\displaystyle \int _{{\mathbb {R}}}\int _{x}^{x+h}| T_{1,2}(x,y)|\cdot |\psi (y)|\mathrm{d}y\mathrm{d}x\\&\quad \le \displaystyle \int _{{\mathbb {R}}}[T_{1,1}(x+h,0) -T_{1,1}(x,0)]\mathrm{d}x\cdot |H(\psi )|\\&\qquad +\displaystyle \int _{0}^{l}\left( \int _{{\mathbb {R}}}[T_{1,2} (x+h,y)-T_{1,2}(x,y)]\mathrm{d}x\right) |\psi (y)|\mathrm{d}y\\&\qquad +\displaystyle \int _{{\mathbb {R}}}\left( \int _{y-h}^{y}| T_{1,2}(x,y)|\mathrm{d}x\right) |\psi (y)|\mathrm{d}y. \end{aligned}$$

The three addends at the right-hand side above vanish as $h\rightarrow 0$ uniformly with respect to $\psi \in U$ thanks to the uniform continuity of *T*. Indeed, the latter also implies from (17) that $|H(\psi )|\le km$ for some positive constant *k*. $\square$

### Proposition 2

(**compactness for model B**) Under (HB1), (HB2) and (HB3), the NGO $BM^{-1}$ defined through (11) and (12) with (13) is compact.

### Proof

(HB2) guarantees that *B* in (11) is bounded. Given $\psi \in X$, let us compute $\phi =M^{-1}\psi \in {\mathcal {D}}(M)$ to show that $M^{-1}$ is compact. By recalling (12) we end up with the IVP

19 $$\begin{aligned} \left\{ \begin{array}{l} \phi '(x)=\displaystyle -\eta (x)\phi (x)+\psi (x),\quad x\in [0,l],\\ \phi (0)=\alpha \end{array}\right. \end{aligned}$$

for some $\alpha \ge 0$ given by imposing the boundary condition characterizing (13), see (21) below.

Now, for any $y\in [0,l]$, let *T*(*x*, *y*) for $x\in [0,l]$, $x\ge y$, be the principal matrix (actually a scalar) solution at *y* of the homogeneous part of (19), i.e.,

20 $$\begin{aligned} T(x,y):=e^{-\displaystyle \int _{y}^{x}\eta (z)\mathrm{d}z}. \end{aligned}$$

Clearly, *T* is uniformly continuous. Moreover, the variation of constants formula gives

$$\begin{aligned} \phi (x)=T(x,0)\alpha +\int _{0}^{x}T(x,y)\psi (y)\mathrm{d}y. \end{aligned}$$

Then it is not difficult to recover

21 $$\begin{aligned} \alpha =H(\psi ;\theta ):=\frac{\displaystyle \theta \int _{0}^{l}\beta (x) \int _{0}^{x}T(x,y)\psi (y)\mathrm{d}y\mathrm{d}x}{\displaystyle 1-\theta \int _{0}^{l} \beta (x)T(x,0)\mathrm{d}x} \end{aligned}$$

through the boundary condition in (13). Note that *H* is well-defined since $T(x,0)\in (0,1)$ for every $x\in (0,l]$, $\theta \in [0,1]$ and (HB1) holds for $\beta$, so that

$$\begin{aligned} 1-\theta \int _{0}^{l}\beta (x)T(x,0)\mathrm{d}x\in (0,1]. \end{aligned}$$

Eventually we get

22 $$\begin{aligned} (M^{-1}\psi )(x)=T(x,0)H(\psi ;\theta )+\int _{0}^{x}T(x,y)\psi (y)\mathrm{d}y. \end{aligned}$$

Compactness of $M^{-1}$ now follows as in the proof of Proposition 1. Indeed, the uniform continuity of *T* and (HB3) give $|H(\psi ;\theta )|\le km$ for some non-negative constant $k=k(\theta )$ (with $k(0)=0$). $\square$

## Discretization and Convergence Analysis

In the first part of this section we give a general overview of discretization and convergence analysis, describing the general structure which is common in treating both model A and model B. Then in the forthcoming Sects. 4.1 and 4.2 we elaborate specifically for the two different classes of models, arriving at proper convergence theorems with error bounds.

As far as discretization is concerned, we resume now the main aspects from [5], where the collocation technique we consider is originally proposed for the sake of numerical testing and application.

Let *N* be a positive integer and $0=:x_{N,0}<x_{N,1}<\cdots <x_{N,N}:=l$ be a mesh of $N+1$ distinct nodes distributed on [0, *l*]. Observe that collocation is meaningless in the whole space $X=L^{1}([0,l],{\mathbb {R}})$ (as is the case for both models A and B). Nevertheless, under the working hypothesis that $R_{0}$ is a generalized eigenvalue (recall (5)), the application of the method is restricted to eigenfunctions $\phi \in {\mathcal {D}}(M)$, which are, in general, smooth enough to guarantee pointwise definition (recall (2)). Moreover, including the nodes $x_{N,0}=0$ and $x_{N,N}=l$ allows for a simplified treatment since for the models of interest in this work the domain ${\mathcal {D}}(M)$ of application is characterized by boundary conditions at one or both the extrema 0, *l* of the domain of the function space *X*, see indeed (9) and (13).

In the sequel, let $X_{N}:={\mathbb {R}}^{N+1}$ be the finite-dimensional counterpart of *X* and let $\varPhi :=(\varPhi _{0},\varPhi _{1},\ldots ,\varPhi _{N})^{T}\in X_{N}$ with $\varPhi _{i}$ representing the numerical approximation of $\phi (x_{N,i})$, $i=0,1,\ldots ,N$. Let us now recall also the *differentiation matrix* and the *quadrature weights* associated to the collocation nodes, as the concerned mortality operators usually involve differentiation and/or integration, see (8) and (12). The first, denoted $H_{N}$, has entries^7^

$$\begin{aligned} h_{N;i,j}:=\ell _{N,j}'(x_{N,i}),\quad i,j=0,1,\ldots ,N, \end{aligned}$$

where $\{\ell _{N,0},\ell _{N,1},\ldots ,\ell _{N,N}\}$ is the Lagrange basis relevant to the chosen nodes: if *f* is a smooth function on [0, *l*] and $v:=(f(x_{N,0}),f(x_{N,1}),\ldots ,f(x_{N,N}))^{T}$, then $H_{N}v$ is an approximation to $(f'(x_{N,0}),f'(x_{N,1}),\ldots , f'(x_{N,N}))^{T}$. The second, components of the vector $w_{N}:=(w_{N,0},w_{N,1},\ldots ,w_{N,N})^{T}\in {\mathbb {R}}^{N+1}$, are given by

23 $$\begin{aligned} w_{N,j}:=\int _{0}^{l}\ell _{N,j}(x)\mathrm{d}x,\quad j=0,1,\ldots ,N, \end{aligned}$$

and, for the same *v* above, $w_{N}^{T}v$ is an approximation to $\int _{0}^{l}f(x)\mathrm{d}x$. Both follow straightforwardly from approximating *f* with the *N*-degree interpolating polynomial

24 $$\begin{aligned} p_{N}(x):=\sum _{j=0}^{N}\ell _{N,j}(x)f(x_{N,j}),\quad x\in [0,l], \end{aligned}$$

which indeed satisfies $p(x_{N,i})=f(x_{N,i})$, $i=0,1,\ldots ,N$. In the sequel we assume the following.

(A3) The collocation nodes $x_{N,0},x_{N,1},\ldots ,x_{N,N}$ are the Chebyshev extrema in [0, *l*], i.e.,
  $$\begin{aligned} x_{N,i}=\frac{l}{2}\left[ 1-\cos \left( \frac{i\pi }{N}\right) \right] ,\quad i=0,1,\ldots ,N. \end{aligned}$$

Under (A3) both $H_{N}$ and $w_{N}$ can be obtained rather easily^8^ (see, e.g., [13, 37, 39]).

The collocation approach reduces the generalized eigenvalue problem (5) to a finite-dimensional discrete version

25 $$\begin{aligned} B_{N}\varPhi =\lambda M_{N}\varPhi \end{aligned}$$

posed on $X_{N}$. The structure of the matrix representation of the finite-dimensional operators $B_{N}$ and $M_{N}$ depends on the specific model as detailed in the following Sects. 4.1 and 4.2. A rigorous error estimation and a related convergence analysis show that the eigenvalues of (25) approximate (part of) the eigenvalues of (5), the accuracy improving as *N* increases. The eigenvalues of (25) can be computed with standard techniques for finite-dimensional generalized eigenvalue problems (e.g., Matlab’s eig, based on the well-known QZ algorithm, see, e.g., [16]). Let us remark that we are mostly concerned with the dominant part of the spectrum, given that we are interested in the spectral radius of the NGO. Let us stress again that the use of the proposed methodology to approximate $R_{0}$ is based on the assumption that this number actually corresponds to a (generalized) eigenvalue. Nevertheless, in [5] as well as in Sect. 5, we report on some tests where the scheme is still able to give reasonable approximations even if $R_{0}$ is not an eigenvalue (not surprisingly the relevant convergence is slower than what proved for eigenvalues).

To study the error between exact and approximated eigenvalues, i.e., those of (5) and (25), respectively, we follow the underlying idea of the approach used in [6], where the analysis relies on the application of Rouché’s Theorem on zeros of holomorphic functions (see, e.g., 7.7 in [31]) to the exact and discrete characteristic equations, say

26 $$\begin{aligned} g(\lambda )=0,\qquad g_{N}(\lambda )=0 \end{aligned}$$

for *g* and $g_{N}$ the exact and discrete characteristic functions, respectively. Both these functions depend on the specific class of models at hand, so that we recover them in the forthcoming Sect. 4.1.2 (model A) and Sect. 4.1.2 (model B) to allow for a separated analysis. In those sections we also show that their difference depends on the collocation error of the associated differential or integro-differential problems. For the time being, to give a general overview of the convergence analysis, let us assume that in some neighborhood of an exact characteristic root $\lambda ^{*}$ to be specified below we can write

27 $$\begin{aligned} |g_{N}(\lambda )-g(\lambda )|\le \varepsilon _{N} \end{aligned}$$

for a quantity $\varepsilon _{N}$ related to the collocation error, and that

28 $$\begin{aligned} \lim _{N\rightarrow \infty }\varepsilon _{N}=0, \end{aligned}$$

possibly under some regularity assumptions on the model coefficients. In the remaining of this section we show that the final error on the eigenvalues depends on this quantity $\varepsilon _{N}$, and thus also that the rate of convergence depends on the convergence of the limit (28).

We first prove the following as a consequence of the exact eigenvalues being isolated with finite multiplicities (following compactness of the NGO, Sect. 3).

### Lemma 1

(**see Lemma 3.5 in** [6]) Let $\lambda ^{*}$ be a zero of *g* with algebraic multiplicity *m*. Then there exists $\rho _{1}=\rho _{1}(\lambda ^{*})>0$ and $C_{1}=C_{1}(\lambda ^{*})>0$ such that for all $\lambda \in \overline{B}(\lambda ^{*},\rho _{1})\setminus \{\lambda ^{*}\}$

$$\begin{aligned} |g(\lambda )|>C_{1}|\lambda -\lambda ^{*}|^{m}. \end{aligned}$$

### Proof

By considering the Taylor series of $g(\lambda )$ around $\lambda ^{*}$ and by taking into account the multiplicity *m* we obtain $g(\lambda )=g^{(m)}(\lambda ^{*})(\lambda -\lambda ^{*})^{m}/m!+O\left( |\lambda -\lambda ^{*}|^{m+1}\right)$ with $g^{(m)}(\lambda ^{*})\ne 0$. Then $\lim _{\lambda \rightarrow \lambda ^{*}}|g(\lambda )|/|\lambda -\lambda ^{*}|^{m}=|g^{(m)}(\lambda ^{*})|m!$. Let us set $C_{1}':=|g^{(m)}(\lambda ^{*})|/m!$. Since $C_{1}'>0$, there exists $\rho _{1}>0$ and $C_{1}>0$, both depending on $\lambda ^{*}$, such that, for all $\lambda \in \overline{B}(\lambda ^{*},\rho _{1})\setminus \{\lambda ^{*}\}$, $|g(\lambda )|/|\lambda -\lambda ^{*}|^{m}>C_{1}$. $\square$

### Theorem 1

(**see Theorem 3.6 in** [6]) Let $\lambda ^{*}$ be a zero of *g* with algebraic multiplicity *m* and $r>0$ be such that $\lambda ^{*}$ is the only zero of *g* in $\overline{B}(\lambda ^{*},r)$. Then there exists $N^{*}\in {\mathbb {N}}$ such that, for all the integers $N\ge N^{*}$, $g_{N}$ has exactly *m* zeros $\lambda _{N,1},\dots ,\lambda _{N,m}$ (counted with multiplicities) in $\overline{B}(\lambda ^{*},r)$ and

$$\begin{aligned} \max _{i=1,\dots ,m}|\lambda ^{*}-\lambda _{N,i}|\le \rho (N), \end{aligned}$$

with $\rho (N)=O(\varepsilon _{N}^{1/m})$ for $\varepsilon _{N}$ in (27).

### Proof

Thanks to Lemma 1 there exist $\rho _{1}=\rho _{1}(\lambda ^{*})$ and $C_{1}=C_{1}(\lambda ^{*})>0$ such that $|g(\lambda )|>C_{1}|\lambda -\lambda ^{*}|^{m}$ for all $\lambda \in \overline{B}(\lambda ^{*},\rho _{1})\setminus \{\lambda ^{*}\}$. We can assume $\rho _{1}<r$ without loss of generality. Let us define $\rho (N):=(\varepsilon _{N}/C_{1})^{1/m}$. Since $\rho (N)\rightarrow 0$ as $N\rightarrow \infty$ thanks to (28), there exists $N^{*}$ sufficiently large such that, for $N\ge N^{*}$, $\rho (N)<\rho _{1}$. Then

$$\begin{aligned} |g(\lambda )|>C_{1}|\lambda -\lambda ^{*}|^{m}=C_{1}\rho (N)^{m} =\varepsilon _{N}\ge |g_{N}(\lambda )-g(\lambda )| \end{aligned}$$

follows by taking $\lambda \in \overline{B}(\lambda ^{*},\rho _{1})$ such that $|\lambda -\lambda ^{*}|= \rho (N)$ and from (27). Since both $g_{N}$ and *g* are holomorphic in $\overline{B}(\lambda ^{*},\rho _{1})$, Rouché’s Theorem (see, e.g., 7.7 in [31]) ensures that they have the same number of zeros in $\overline{B}(\lambda ^{*},\rho (N))$ counted with multiplicities. $\square$

In the next sections we recover both (26) and (27) for either the class of cell population models (model A) and that of epidemic models (model B). We show also that (28) holds in both cases, giving account of the relevant rate of convergence, thus re-elaborating Theorem 1 into Theorem 2 and Theorem 3 for the two specific classes of models.

### Model A

#### Discretization

Let us recall the main ingredients (7), (8) and (9) of the class of cell population models, model A in Sect. 2.1, as well as the generalized eigenvalue problem (5) with generalized eigenfunction $\phi \in {\mathcal {D}}(M)\setminus \{0\}$. Since we are interested in the spectral radius, we assume $\lambda \ne 0$. Therefore, by using (15) we can combine (5) and (8) to get

29 $$\begin{aligned} \frac{1}{\lambda }B\phi =M\phi =\xi '+(\beta +\mu )\phi . \end{aligned}$$

Then, similarly to the proof of Proposition 1, we arrive at the first-order system of nonautonomous ODEs

$$\begin{aligned} \begin{pmatrix} \phi '\\ \xi ' \end{pmatrix} =A^{(\lambda )} \begin{pmatrix} \phi \\ \xi \end{pmatrix} \end{aligned}$$

for

30 $$\begin{aligned} A^{(\lambda )}:=\begin{pmatrix} \displaystyle \frac{c}{D}&{}\displaystyle -\frac{1}{D}\\ \displaystyle \frac{2\beta }{\lambda }-\beta -\mu &{}0 \end{pmatrix}, \end{aligned}$$

which is well-defined under (HA3). Consider the associated IVP

31 $$\begin{aligned} \left\{ \begin{array}{l} \begin{pmatrix} \phi '(x)\\ \xi '(x) \end{pmatrix} =A^{(\lambda )}(x) \begin{pmatrix} \phi (x)\\ \xi (x) \end{pmatrix} ,\quad x\in [0,l],\\ \begin{pmatrix} \phi (0)\\ \xi (0) \end{pmatrix} = \begin{pmatrix} \alpha \\ \omega \end{pmatrix} \end{array}\right. \end{aligned}$$

for some $(\alpha ,\omega )^{T}\in {\mathbb {R}}^{2}$. The discrete generalized eigenvalue problem (25) is obtained by collocating (31) and by imposing the boundary conditions

32 $$\begin{aligned} \xi (0)=0=\xi (l) \end{aligned}$$

characterizing ${\mathcal {D}}(M)$. Therefore we look for *N*-degree polynomials $p_{N}$ and $q_{N}$ satisfying

33 $$\begin{aligned} \left\{ \begin{array}{l} \begin{pmatrix} p_{N}'(x_{N,i})\\ q_{N}'(x_{N,i}) \end{pmatrix} =A^{(\lambda )}(x_{N,i}) \begin{pmatrix} p_{N}(x_{N,i})\\ q_{N}(x_{N,i}) \end{pmatrix} ,\quad i=1,\ldots ,N,\\ \begin{pmatrix} p_{N}(0)\\ q_{N}(0) \end{pmatrix} = \begin{pmatrix} \alpha \\ \omega \end{pmatrix} \end{array}\right. \end{aligned}$$

together with

34 $$\begin{aligned} q_{N}(0)=0=q_{N}(l). \end{aligned}$$

By setting

35 $$\begin{aligned} p_{N}(x)=\sum _{j=0}^{N}\ell _{N,j}(x)\varPhi _{j},\qquad q_{N}(x) =\sum _{j=0}^{N}\ell _{N,j}(x)\varXi _{j} \end{aligned}$$

for $x\in [0,l]$, it is not difficult to recover from the second ODE in (33)

$$\begin{aligned} \frac{2}{\lambda }\beta (x_{N,i})\varPhi _{i}=[H_{N}\varXi ]_{i} +[\beta (x_{N,i})+\mu (x_{N,i})]\varPhi _{i},\quad i=1,\ldots ,N, \end{aligned}$$

for $H_N$ the differentiation matrix given in Sect. 4 and $\varXi :=(\varXi _{0},\varXi _{1},\ldots ,\varXi _{N})^{T}\in X_{N}$ defined as

36 $$\begin{aligned} \varXi _{0}:=\omega \end{aligned}$$

and

37 $$\begin{aligned} \varXi _{i}:=c(x_{N,i})\varPhi _{i}-D(x_{N,i})[H_{N}\varPhi ]_{i},\quad i=1,\ldots ,N, \end{aligned}$$

the latter representing the discrete counterpart of (15), following the first ODE in (33) and with $\varPhi :=(\varPhi _{0},\varPhi _{1},\ldots ,\varPhi _{N})^{T}\in X_{N}$. The boundary conditions (34) translate into

38 $$\begin{aligned} \varXi _{0}=0=\varXi _{N}, \end{aligned}$$

amounting to choose $\omega =0$ and

39 $$\begin{aligned} c(x_{N,N})\varPhi _{N}-D(x_{N,N})[H_{N}\varPhi ]_{N}=0. \end{aligned}$$

Consequentely, by defining the matrices (empty entries are zeros)

40 $$\begin{aligned} B_{N}&:=\begin{pmatrix} 0&{}&{}&{}\\ &{}2\beta (x_{N,1})&{}&{}\\ &{}&{}\ddots &{}\\ &{}&{}&{}2\beta (x_{N,N}) \end{pmatrix}\in {\mathbb {R}}^{(N+1)\times (N+1)},\nonumber \\ C_{N}&:= \begin{pmatrix} c(x_{N,0})&{}&{}\\ &{}\ddots &{}\\ &{}&{}c(x_{N,N}) \end{pmatrix}\in {\mathbb {R}}^{(N+1)\times (N+1)},\nonumber \\ D_{N}&:=\begin{pmatrix} D(x_{N,0})&{}&{}\\ &{}\ddots &{}\\ &{}&{}D(x_{N,N}) \end{pmatrix}\in {\mathbb {R}}^{(N+1)\times (N+1)} \end{aligned}$$

and

$$\begin{aligned} \varSigma _{N}:=\begin{pmatrix} \beta (x_{N,0})+\mu (x_{N,0})&{}&{}\\ &{}\ddots &{}\\ &{}&{}\beta (x_{N,N})+\mu (x_{N,N}) \end{pmatrix}\in {\mathbb {R}}^{(N+1)\times (N+1)} \end{aligned}$$

we obtain (25) with $M_{N}$ defined as

41 $$\begin{aligned} M_{N;0,0:N}:=c(x_{N,N})\delta _{N,0:N}-D(x_{N,N})H_{N;N,0:N} \end{aligned}$$

for $\delta _{i,j}$ the Kronecker’s delta and

42 $$\begin{aligned} M_{N;1:N,0:N}:=H_{N;1:N,1:N}(C_{N;1:N,0:N}-D_{N;1:N,0:N}H_{N})+\varSigma _{N;1:N,0:N}. \end{aligned}$$

Let us remark that the first row of $M_{N}$ defined in (41) accounts for the boundary condition $\varXi _{N}=0$ through (39) thanks to the first trivial row of $B_{N}$ in (40). The other boundary condition $\varXi _{0}=0$ is hidden in removing the first column of $H_{N}$ in the first factor of the product giving the first addend at the right-hand side of (42), which implicitly corresponds to annihilate the first row of $C_{N}-D_{N}H_{N}$ and thus imposing

$$\begin{aligned} c(x_{N,0})\varPhi _{0}-D(x_{N,0})[H_{N}\varPhi ]_{0}=0, \end{aligned}$$

i.e., extending definition (37) to the index $i=0$ and choosing $\omega =0$ in accordance with (36).

Finally, observe that the parameter $\alpha$ is left free, which indeed amounts to the degree of freedom of the generalized eigenfunctions due to parallelism.

##### Remark 1

Let us emphasize that the above approach is slightly different from the one originally proposed in [5, Section 3.1]. Indeed, therein the discretization was obtained by collocation of the generalized eigenvalue problem (5), while here collocation is applied to the corresponding IVP (31). Although the two alternatives are numerically equivalent (in the sense that the outcome of the numerical experiments is practically indistinguishable, see (T1A) in Sect. 5), the current one is more favorable in terms of its convergence analysis. In fact, working with IVPs, rather than with Boundary Value Problems (BVPs), easily leads to the formulation of an exact characteristic equation as carried out in the sequel, as well as of that of a numerical counterpart.

#### Exact and Discrete Characteristic Equations

The first step of the convergence analysis consists in finding a characteristic equation for the generalized eigenvalue problem (5). To this aim, let $T^{(\lambda )}:[0,l]\rightarrow {\mathbb {R}}^{2\times 2}$ be the principal matrix solution at 0 of the IVP (31), i.e., the matrix solution of

$$\begin{aligned} \left\{ \begin{array}{l} \displaystyle {T^{(\lambda )}}'(x)= A^{(\lambda )}(x)T^{(\lambda )}(x),\quad x\in [0,l],\\ T^{(\lambda )}(0)=I_{2}, \end{array}\right. \end{aligned}$$

so that

43 $$\begin{aligned} \begin{pmatrix} \phi (x)\\ \xi (x) \end{pmatrix} =T^{(\lambda )}(x) \begin{pmatrix} \alpha \\ \omega \end{pmatrix}, \quad x\in [0,l], \end{aligned}$$

is the solution of (31). By choosing $x=l$, the application of the boundary conditions (32) leads to

$$\begin{aligned} 0=\xi (l)=T^{(\lambda )}_{2,1}(l)\alpha +T^{(\lambda )}_{2,2}(l) \omega =T^{(\lambda )}_{2,1}(l)\alpha . \end{aligned}$$

Therefore, the sought characteristic equation reads

44 $$\begin{aligned} g(\lambda )=0 \end{aligned}$$

for the characteristic function

45 $$\begin{aligned} g(\lambda ):=T_{2,1}^{(\lambda )}(l). \end{aligned}$$

Observe that *g* is well-defined but not known explicitly, this lack having no consequences in the sequel.

For later use, note that (43) can be equivalently characterized as the solution of the functional equation in $X^{2}$

46 $$\begin{aligned} \begin{pmatrix} \phi \\ \xi \end{pmatrix} =\begin{pmatrix} \alpha \\ \omega \end{pmatrix} +VA^{(\lambda )} \begin{pmatrix} \phi \\ \xi \end{pmatrix}, \end{aligned}$$

where $V:X^{2}\rightarrow X^{2}$ is the Volterra integral operator

$$\begin{aligned} (Vu)(x):=\int _{0}^{x}u(y)\mathrm{d}y,\quad x\in [0,l]. \end{aligned}$$

Let us now recover the discrete version of the characteristic equation (44). Assuming existence and uniqueness of the collocation solution $(p_{N},q_{N})^{T}$ of (33) (see Proposition 3 below), we can define $T^{(\lambda )}_{N}:[0,l]\rightarrow {\mathbb {R}}^{2\times 2}$ such that

47 $$\begin{aligned} \begin{pmatrix} p_{N}(x)\\ q_{N}(x) \end{pmatrix} =T^{(\lambda )}_{N}(x) \begin{pmatrix} \alpha \\ \omega \end{pmatrix}, \quad x\in [0,l], \end{aligned}$$

i.e., the discrete counterpart of $T^{(\lambda )}(x)$ in (43). Then (38) and the second of (35) necessarily lead to

$$\begin{aligned} 0=\varXi _{N}=T^{(\lambda )}_{N;2,1}(l)\alpha +T^{(\lambda )}_{N;2,2}(l) \omega =T^{(\lambda )}_{N;2,1}(l)\alpha . \end{aligned}$$

Therefore, the sought discrete characteristic equation reads

48 $$\begin{aligned} g_{N}(\lambda )=0 \end{aligned}$$

for the discrete characteristic function

49 $$\begin{aligned} g_{N}(\lambda ):=T^{(\lambda )}_{N;2,1}(l). \end{aligned}$$

Finally, and again for later use, we can recover the discrete version of the functional Eq. (46). Indeed, since $p_{N},q_{N}$ are *N*-degree polynomials, it is clear that their first derivative is interpolated exactly at *N* nodes, so that we can write

$$\begin{aligned} \begin{pmatrix} p_{N}(x)\\ q_{N}(x) \end{pmatrix}= & {} \displaystyle \begin{pmatrix} \alpha \\ \omega \end{pmatrix} +\int _{0}^{x} \begin{pmatrix} p_{N}'(y)\\ q_{N}'(y) \end{pmatrix} \mathrm{d}y\\= & {} \displaystyle \begin{pmatrix} \alpha \\ \omega \end{pmatrix} +\int _{0}^{x}\sum _{j=1}^{N}\ell _{N-1,j}(y) \begin{pmatrix} p_{N}'(x_{N,j})\\ q_{N}'(x_{N,j}) \end{pmatrix} \mathrm{d}y\\= & {} \displaystyle \begin{pmatrix} \alpha \\ \omega \end{pmatrix} +\int _{0}^{x}\sum _{j=1}^{N}\ell _{N-1,j}(y)A^{(\lambda )}(x_{N,j}) \begin{pmatrix} p_{N}(x_{N,j})\\ q_{N}(x_{N,j}) \end{pmatrix} \mathrm{d}y\\ \end{aligned}$$

for $\{\ell _{N-1,1},\ldots ,\ell _{N-1,N}\}$ the Lagrange basis relevant to the nodes $x_{N,1},\ldots ,x_{N,N}$. If ${\mathcal {L}}_{N-1}:X^{2}\rightarrow X^{2}$ is the relevant Lagrange interpolation operator, we obtain

50 $$\begin{aligned} \begin{pmatrix} p_{N}\\ q_{N} \end{pmatrix} =\begin{pmatrix} \alpha \\ \omega \end{pmatrix} +V{\mathcal {L}}_{N-1}A^{(\lambda )} \begin{pmatrix} p_{N}\\ q_{N} \end{pmatrix}. \end{aligned}$$

Note that the possibility of obtaining both (46) and (50) is the advantage of working with IVPs mentioned in Remark 1.

#### Collocation Error and Convergence

Let us define from (31) and (33) the collocation error

$$\begin{aligned} e_{N}:=\begin{pmatrix} p_{N}\\ q_{N} \end{pmatrix} -\begin{pmatrix} \phi \\ \xi \end{pmatrix} \in X^{2}. \end{aligned}$$

If we let

$$\begin{aligned} {\mathcal {E}}_{N}(x):=T^{(\lambda )}_{N}(x)-T^{(\lambda )}(x),\qquad x\in [0,l], \end{aligned}$$

then

$$\begin{aligned} \Vert {\mathcal {E}}_{N}(x)\Vert =\sup _{(\alpha ,\omega )^{T}\in {\mathbb {R}}^{2} \setminus \{(0,0)^{T}\}}\frac{\left\| e_{N}(x)\right\| }{\Vert (\alpha ,\omega )^{T}\Vert } \end{aligned}$$

follows from (43) and (47). Since the exact and the discrete characteristic functions are defined by (45) and (49), it follows that

51 $$\begin{aligned} |g_{N}(\lambda )-g(\lambda )|\le \sup _{(\alpha ,\omega )^{T} \in {\mathbb {R}}^{2}\setminus \{(0,0)^{T}\}}\frac{\left\| e_{N}(l)\right\| }{\Vert (\alpha ,\omega )^{T}\Vert }. \end{aligned}$$

It is then clear that we need a bound for $\Vert e_{N}(l)\Vert$, and this is the goal of the analysis in this section.

By subtracting (46) from (50) we get the functional equation for the collocation error in $X^{2}$

52 $$\begin{aligned} e_{N}=V{\mathcal {L}}_{N-1}A^{(\lambda )}e_{N}+Vr_{N} \end{aligned}$$

with

53 $$\begin{aligned} r_{N}:=({\mathcal {L}}_{N-1}-I_{X^{2}})A^{(\lambda )} \begin{pmatrix} \phi \\ \xi \end{pmatrix} \end{aligned}$$

for $(\phi ,\xi )^{T}$ the solution of (31). We are now able to prove the following result about the existence and uniqueness of the collocation error as *the* solution of (52) for *N* large enough. Of course, this in turn implies also the existence and uniqueness of the collocation solution $(p_{N},q_{N})^{T}$ and thus it gives a proper sense to the discrete characteristic equation (48) as recovered in Sect. 4.1.2.

##### Proposition 3

Under (HA3), (HA4) and (A3), there exists $N^{*}\in {\mathbb {N}}$ such that for all the integers $N\ge N^{*}$ (52) has a unique solution $e_{N}$ and

54 $$\begin{aligned} \Vert e_{N}(l)\Vert \le 2\left\| \left( I_{X^{2}}-A^{(\lambda )}V\right) ^{-1}\right\| _{X^{2} \leftarrow X^{2}}\Vert r_{N}\Vert _{X^{2}}. \end{aligned}$$

##### Proof

Besides (52) let us consider also the functional equation in $X^{2}$

55 $$\begin{aligned} \tilde{e}_{N}={\mathcal {L}}_{N-1}A^{(\lambda )}V\tilde{e}_{N}+r_{N}. \end{aligned}$$

It is immediate to verify that the solutions of (52) are one-to-one with those of (55) through $e_{N}=V\tilde{e}_{N}$ and $\tilde{e}_{N}={\mathcal {L}}_{N-1}A^{(\lambda )}e_{N}+r_{N}$. We prove then that (55) admits a unique solution for *N* large enough by showing through the Banach’s perturbation lemma (see, e.g., Theorem 10.1 in [24]) that $I_{X^{2}}-{\mathcal {L}}_{N-1}A^{(\lambda )}V$ is invertible with (uniformly) bounded inverse. To this aim we need to prove that (a) $I_{X^{2}}-A^{(\lambda )}V$ is invertible with bounded inverse and that (b) $\Vert ({\mathcal {L}}_{N-1}-I_{X^{2}})A^{(\lambda )}V\Vert _{X^{2} \leftarrow X^{2}}$ vanishes as $N\rightarrow \infty$. (a) follows since $\eta '=A^{(\lambda )}\eta +\psi$ has a unique solution $\eta$ for any given $\psi \in X^{2}$, so that by letting $\varphi :=\eta '$ we have $\eta =V\varphi$ and $\varphi =A^{(\lambda )}V\varphi +\psi$. As for (b) it is enough to observe that $V(X^{2})$ is a subset of the space $C^{2}\subset X^{2}$ of continuous functions, and so is $A^{(\lambda )}V(X^{2})$ under (HA3) and (HA4) through (30). Then (b) follows since under (A3) $\Vert {\mathcal {L}}_{N-1}-I_{X^{2}}\Vert _{X^{2}\leftarrow C^{2}}$ vanishes as $N\rightarrow \infty$ thanks to Corollary of Theorem Ia in [14]. Then

$$\begin{aligned} \Vert \tilde{e}_{N}\Vert _{X^{2}}\le 2\left\| \left( I_{X^{2}} -A^{(\lambda )}V\right) ^{-1}\right\| _{X^{2}\leftarrow X^{2}}\Vert r_{N}\Vert _{X^{2}} \end{aligned}$$

follows for *N* large enough since the Banach’s perturbation lemma gives also

$$\begin{aligned} \left\| \left( I_{X^{2}}-{\mathcal {L}}_{N-1}A^{(\lambda )}V\right) ^{-1}\right\| _{X^{2} \leftarrow X^{2}}\le 2\left\| \left( I_{X^{2}}-A^{(\lambda )} V\right) ^{-1}\right\| _{X^{2}\leftarrow X^{2}}. \end{aligned}$$

The thesis is eventually obtained by observing that $e_{N}=V\tilde{e}_{N}$ gives indeed $e_{N}(l)=\int _{0}^{l}\tilde{e}_{N}(x)\mathrm{d}x$. $\square$

##### Remark 2

Note that the continuity hypothesis (HA4) on *c*, *D*, $\beta$ and $\mu$ can be further weakened. Indeed, point (a) in the proof above is guaranteed in the class of Lebesgue integrable functions (Carathéodory’s existence theorem for ODEs, see, e.g., [10, Chapter 2, Theorem 1.1]), while the validity of Corollary of Theorem Ia in [14] for point (b) is ensured for Riemann integrable functions. In any case, in view of the smoothness required below, such a refinement is useless.

Now, in view of (54), by considering (51) and with reference to (27) in the general analysis at the beginning of Sect. 4, we aim at showing that there exists $\varepsilon _{N}$ vanishing as $N\rightarrow \infty$ such that (56) below holds. Thus we have to evaluate the remainder $r_{N}$ as defined in (53), i.e., the interpolation error on the derivative of the solution of the IVP (31). As such it basically depends on the smoothness of the map $x\mapsto A^{(\lambda )}(x)$ (and thus on that of *c*, *D*, $\beta$ and $\mu$), which determines the growth of the derivatives of the solution $(\phi ,\xi )^{T}$ of (31).

##### Proposition 4

Under (HA3), (HA5) and (A3), there exists $N^{*}\in {\mathbb {N}}$ such that for all the integers $N\ge N^{*}$

56 $$\begin{aligned} \Vert r_{N}\Vert _{X^{2}}\le \varepsilon _{N}\left\| \begin{pmatrix} \alpha \\ \omega \end{pmatrix}\right\| \end{aligned}$$

with

$$\begin{aligned} \varepsilon _{N}={\left\{ \begin{array}{ll} O(N^{-s}\log N) &{} \text {under (HA5.1)} \\ O(N^{-r}\log N) &{} \text {for every integer}\ r\ge 1\ \text {under (HA5.2)}\\ O(k^{-N}\log N) &{} \text {for some constant}\ k>1\ \text {under (HA5.3).} \end{array}\right. } \end{aligned}$$

##### Proof

In this proof we set $\eta :=(\phi ,\xi )^{T}$ for brevity. From (53) we have

$$\begin{aligned} \left\| ({\mathcal {L}}_{N-1}-I_{X^{2}})A^{(\lambda )}\eta \right\| _{X^{2}} \le l\left\| ({\mathcal {L}}_{N-1}-I_{X^{2}})A^{(\lambda )}\eta \right\| _{\infty }. \end{aligned}$$

As a classical result in uniform approximation we have

$$\begin{aligned} \left\| ({\mathcal {L}}_{N-1}-I_{X^{2}})A^{(\lambda )} \eta \right\| _{\infty }\le (1+\varLambda _{N-1})E_{N-1}\left( A^{(\lambda )}\eta \right) , \end{aligned}$$

where $\varLambda _{N-1}$ is the Lebesgue constant relevant to the nodes $x_{N,1},\ldots ,x_{N,N}$ and $E_{N-1}(f)$ is the best uniform approximation error of a function *f* in $\varPi _{N-1}$ (the set of algebraic polynomials of degree $\le N-1$). As for the former, (A3) guarantees that $\varLambda _{N-1}=O(\log N)$ (see, e.g., Section 4.2.2, page 257 of [29]). As for the latter, under (HA5.1) the map $x\mapsto A^{(\lambda )}(x)$ is of class $C^{s}$ for some integer $s\ge 1$, then so is $A^{(\lambda )}\eta$ and Jackson’s theorem (see, e.g., Section 1.1.2 of [33]) provides

$$\begin{aligned} E_{N-1}\left( A^{(\lambda )}\eta \right) \le c \frac{\left\| \left[ A^{(\lambda )}\eta \right] ^{(s)}\right\| _{\infty }}{N^{s}} \end{aligned}$$

for some constant *c* independent of *N*. The same reasoning holds under (HA5.2). Under (HA5.3) Jackson’s theorem gives an exponential decay for the best uniform approximation error, i.e.,

$$\begin{aligned} E_{N-1}\left( A^{(\lambda )}\eta \right) \le ce^{-\gamma N} \end{aligned}$$

for positive constants *c* and $\gamma$ (see, e.g., page 2783 of [28]), then set $k:=e^{\gamma }$. The final bound follows by considering that $\eta$ depends linearly on $(\alpha ,\omega )^{T}$, recall (43). $\square$

We conclude with the main result for the class of cell population models (model A), direct consequence of Theorem 1 and Proposition 4.

##### Theorem 2

Let $\lambda ^{*}$ be a zero of *g* in (45) with algebraic multiplicity *m* and $r>0$ be such that $\lambda ^{*}$ is the only zero of *g* in $\overline{B}(\lambda ^{*},r)$. Then, under (HA3), (HA5) and (A3), there exists $N^{*}\in {\mathbb {N}}$ such that, for all the integers $N\ge N^{*}$, $g_{N}$ in (49) has exactly *m* zeros $\lambda _{N,1},\dots ,\lambda _{N,m}$ (counted with multiplicities) in $\overline{B}(\lambda ^{*},r)$ and

$$\begin{aligned} \max _{i=1,\dots ,m}|\lambda ^{*}-\lambda _{N,i}|\le \rho (N) \end{aligned}$$

with

$$\begin{aligned} \rho _{N}={\left\{ \begin{array}{ll} O((N^{-s}\log N)^{1/m}) &{}\text {under (HA5.1)} \\ O((N^{-r}\log N)^{1/m}) &{}\text {for every integer}\ r\ge 1\ \text {under (HA5.2)}\\ O((k^{-N}\log N)^{1/m}) &{}\text {for some constant}\ k>1\ \text {under (HA5.3).} \end{array}\right. } \end{aligned}$$

### Model B

#### Discretization

Let us recall the main ingredients (11), (12) and (13) of the class of epidemic models, model B in Sect. 2.2, as well as the generalized eigenvalue problem (5) with generalized eigenfunction $\phi \in {\mathcal {D}}(M)\setminus \{0\}$. Again we are interested in the spectral radius, so we assume $\lambda \ne 0$. Therefore, we can combine (5) and (8) to get

$$\begin{aligned} \frac{1}{\lambda }B\phi =M\phi =\phi '+\eta \phi , \end{aligned}$$

corresponding to the IVP

57 $$\begin{aligned} \left\{ \begin{array}{l} \phi '(x)=\displaystyle -\eta (x)\phi (x)+\frac{1}{\lambda }(B\phi )(x),\quad x\in [0,l],\\ \phi (0)=\displaystyle \theta \int _{0}^{l}\beta (x)\phi (x)\mathrm{d}x. \end{array}\right. \end{aligned}$$

Now, to get (25), we look for an *N*-degree polynomial $p_{N}$ satisfying

58 $$\begin{aligned} \left\{ \begin{aligned} p_{N}'(x_{N,i})&=-\eta (x_{N,i})p_{N}(x_{N,i}) \\&\quad +\displaystyle \frac{1}{\lambda }\sum \limits _{j=0}^{N} w_{N,j}K(x_{N,i},x_{N,j})p_{N}(x_{N,j}),\quad i=1,\ldots ,N,\\ p_{N}(0)&=\theta \sum \limits _{j=0}^{N}w_{N,j}\beta (x_{N,j})p_{N}(x_{N,j}). \end{aligned}\right. \end{aligned}$$

The first part corresponds to the collocation of the first-order nonautonomous ODE in (57) at the nodes $x_{N,1},\ldots ,x_{N,N}$, together with the quadrature of the integral defining the action of *B* in (11) according to the formula introduced in Sect. 4. The second part corresponds to imposing the boundary condition in (57), where again the relevant integral is substituted by the quadrature formula. By setting

$$\begin{aligned} p_{N}(x)=\sum _{j=0}^{N}\ell _{N,j}(x)\varPhi _{j},\quad x\in [0,l], \end{aligned}$$

it is not difficult to recover (25) by defining the matrices $B_{N}\in {\mathbb {R}}^{(N+1)\times (N+1)}$ as

$$\begin{aligned} B_{N,i,j}:=\left\{ \begin{array}{lll} 0,&{}\quad i=0,&{}\;j=0,1,\ldots ,N,\\ w_{N,j}K(x_{N,i},x_{N,j}),&{}\quad i=1,\ldots ,N,&{}\;j=0,1,\ldots ,N, \end{array}\right. \end{aligned}$$

and $$M_{N}\in {\mathbb {R}}^{(N+1)\times (N+1)}$$ as

$$\begin{aligned} M_{N;i,j}:=\left\{ \begin{array}{lll} \delta _{0,j}-\theta w_{N,j}\beta (x_{N,j}),&{}\quad i=0,&{}\;j=0,1,\ldots ,N,\\ H_{N;i,j}+\eta (x_{N,i})\delta _{i,j},&{}\quad i=1,\ldots ,N,&{}\;j=0,1,\ldots ,N. \end{array}\right. \end{aligned}$$

Let us remark that this is exactly the approach presented in [5]: indeed, being already based on an IVP, there is no need for modification in view of the analysis of convergence as for model A (recall Remark 1).

#### Exact and Discrete Characteristic Equations

The equation in (57) is integro-differential, given the integral nature of the birth operator *B* in (11). In view of obtaining a characteristic equation for the generalized eigenvalue problem (5), let us consider the related IVP

59 $$\begin{aligned} \left\{ \begin{array}{l} \phi '(x)=\displaystyle -\eta (x)\phi (x)+\frac{1}{\lambda }(B\phi )(x),\quad x\in [0,l],\\ \phi (\bar{x})=\alpha \end{array}\right. \end{aligned}$$

for some $\bar{x}\in [0,l]$ and $\alpha \in {\mathbb {R}} \setminus \{0\}$. Note that the existence of such a point $\bar{x}$ is guaranteed since $\phi$ is nontrivial being a generalized eigenfunction and, moreover, pointwise evaluation makes sense since functions in ${\mathcal {D}}(M)$ are (absolutely) continuous. In particular, we show next that (59) is well-posed if and only if $\bar{x}\in (0,l]$. Let us also emphasize that this result is independent of $\theta$ in (57), as the latter does not appear in (59).

##### Proposition 5

Under (HB1) and (HB2) (59) has a unique solution, which depends linearly on $\alpha$, if and only if $\bar{x}\in (0,l]$.

##### Proof

The variation of constants formula as applied to the linear nonautonomous and inhomogeneous ODE in (59) yields

60 $$\begin{aligned} \phi (x)=T(x,\bar{x})\alpha +\int _{\bar{x}}^{x}T(x,y) \frac{1}{\lambda }(B\phi )(y)\mathrm{d}y \end{aligned}$$

for *T* in (20). (60) becomes the Fredholm integral equation of second kind in *X*

$$\begin{aligned} \phi =f+L\phi \end{aligned}$$

by defining

61 $$\begin{aligned} f:[0,l]\rightarrow {\mathbb {R}},\qquad f(x):=T(x,\bar{x})\alpha , \end{aligned}$$

and

62 $$\begin{aligned} L:X\rightarrow X,\qquad (L\varphi )(x):=\int _{\bar{x}}^{x}T(x,y) \frac{1}{\lambda }(B\varphi )(y)\mathrm{d}y,\quad x\in [0,l]. \end{aligned}$$

Under (HB1) and (HB2) *L* is compact (Proposition 9), therefore the thesis on existence and uniqueness follows from the relevant Riesz theory (see, in particular, Corollary 3.5 in [24]). Indeed, under (HB1) $I_{X}-L$ is injective if and only if $\bar{x}\in (0,l]$ (Proposition 10). The linearity with respect to $\alpha$ is clear from (61). $\square$

Again, as observed at the end of Sect. 4.1, the parameter $\alpha$ is left free since $\phi$ is a generalized eigenfunction. Note, moreover, that the IVP is well-posed also if $\alpha =0$ when $\bar{x}\in (0,l]$. Indeed, it would not be well-posed for $\alpha =0$ only if $\bar{x}=0$, given that in this case the trivial solution would always exist (due to the linearity of the problem) beyond a nontrivial eigenfunction of the corresponding generalized eigenvalue problem (5) in the case $\theta =0$.

Thanks to Proposition 5, fix $\bar{x}\in (0,l]$ and let $T^{(\lambda )}(\cdot ,\bar{x}):[0,l]\rightarrow {\mathbb {R}}$ be such that

63 $$\begin{aligned} \phi (x)=T^{(\lambda )}(x,\bar{x})\alpha ,\quad x\in [0,l], \end{aligned}$$

is the unique solution of (59). The application of the boundary condition in (57) characterizing ${\mathcal {D}}(M)$ in (13) leads to

$$\begin{aligned} T^{(\lambda )}(0,\bar{x})\alpha =\theta \int _{0}^{l}\beta (x) T^{(\lambda )}(x,\bar{x})\alpha \mathrm{d}x. \end{aligned}$$

Therefore, the sought characteristic equation reads

64 $$\begin{aligned} g(\lambda )=0 \end{aligned}$$

for the characteristic function

65 $$\begin{aligned} g(\lambda ):=T^{(\lambda )}(0,\bar{x})-\theta \int _{0}^{l} \beta (x)T^{(\lambda )}(x,\bar{x})\mathrm{d}x. \end{aligned}$$

Note that, similarly to (45), *g* is well-defined but not known explicitly, since so is $T^{(\lambda )}$. Also, there is no need to specify $\bar{x}$ as its existence is enough to define *g*.

##### Remark 3

Note that if $\bar{x}=0$ then

$$\begin{aligned} g(\lambda )=1-\theta \int _{0}^{l}\beta (x)T^{(\lambda )}(x,0)\mathrm{d}x, \end{aligned}$$

which never vanishes when $\theta =0$.

For later use, note that (63) can be equivalently characterized as the solution of the functional equation in *X*

66 $$\begin{aligned} \phi =\alpha +VA^{(\lambda )}\phi , \end{aligned}$$

where $V:X\rightarrow X$ is the Volterra integral operator

67 $$\begin{aligned} (V\xi )(x):=\int _{\bar{x}}^{x}\xi (y)\mathrm{d}y,\quad x\in [0,l], \end{aligned}$$

and $A^{(\lambda )}:X\rightarrow X$ is the linear operator giving the right-hand side of the equation in the IVP (59), i.e.,

68 $$\begin{aligned} A^{(\lambda )}\xi :=-\eta \xi +\frac{1}{\lambda }B\xi . \end{aligned}$$

Let us now recover the discrete version of the characteristic equation (64). Let $p_{N}$ be the collocation solution of (59), i.e.,

69 $$\begin{aligned} \left\{ \begin{aligned} p_{N}'(x_{N,i})&=-\eta (x_{N,i})p_{N}(x_{N,i})\\&\quad +\displaystyle \frac{1}{\lambda }\sum \limits _{j=0}^{N} w_{N,j}K(x_{N,i},x_{N,j})p_{N}(x_{N,j}),\quad i=1,\ldots ,N,\\ p_{N}(\bar{x})&=\alpha , \end{aligned}\right. \end{aligned}$$

whose existence and uniqueness is proved in Proposition 6 below. We can define $T^{(\lambda )}_{N}(\cdot ,\bar{x}):[0,l]\rightarrow {\mathbb {R}}$ such that

70 $$\begin{aligned} p_{N}(x)=T^{(\lambda )}_{N}(x,\bar{x})\alpha ,\quad x\in [0,l], \end{aligned}$$

i.e., the discrete counterpart of $T^{(\lambda )}$ in (63). Then the second of (58) and (70) necessarily lead to

$$\begin{aligned} T^{(\lambda )}_{N}(0,\bar{x})\alpha =\theta \sum _{j=0}^{N}w_{N,j} \beta (x_{N,j})T^{(\lambda )}_{N}(x_{N,j},\bar{x})\alpha . \end{aligned}$$

Therefore, the sought discrete characteristic equation reads

$$\begin{aligned} g_{N}(\lambda )=0 \end{aligned}$$

for the discrete characteristic function

71 $$\begin{aligned} g_{N}(\lambda ):=T^{(\lambda )}_{N}(0,\bar{x})-\theta \sum _{j=0}^{N}w_{N,j} \beta (x_{N,j})T^{(\lambda )}_{N}(x_{N,j},\bar{x}). \end{aligned}$$

Finally, and again for later use, we recover the discrete version of the functional equation (66), as done in Sect. 4.1.2 for (50). It reads

72 $$\begin{aligned} p_{N}=\alpha +V{\mathcal {L}}_{N-1}A^{(\lambda )}_{N}p_{N}, \end{aligned}$$

where *V* is given in (67), ${\mathcal {L}}_{N-1}:X\rightarrow X$ is the Lagrange interpolation operator relevant to the nodes $x_{N,1},\ldots ,x_{N,N}$ and the quadrature approximation $A^{(\lambda )}_{N}:X\rightarrow X$ given by

$$\begin{aligned} (A^{(\lambda )}_{N}\xi )(x):=-\eta (x)\xi (x)+\frac{1}{\lambda } \sum _{j=0}^{N}w_{N,j}K(x,x_{N,j})\xi (x_{N,j}),\quad x\in [0,l], \end{aligned}$$

replaces $$A^{(\lambda )}$$ in (68).

#### Quadrature and Collocation Errors, and Convergence

In view of (27), from (65) and (71) we have

73 $$\begin{aligned} g_{N}(\lambda )-g(\lambda )&=\displaystyle T^{(\lambda )}_{N}(0,\bar{x}) -\theta \sum _{j=0}^{N}w_{N,j}\beta (x_{N,j})T^{(\lambda )}_{N}(x_{N,j},\bar{x})\nonumber \\&\quad -\displaystyle T^{(\lambda )}(0,\bar{x})+\theta \int _{0}^{l}\beta (x) T^{(\lambda )}(x,\bar{x})\mathrm{d}x\nonumber \\&=\displaystyle T^{(\lambda )}_{N}(0,\bar{x})-T^{(\lambda )}(0,\bar{x})\nonumber \\&\quad +\displaystyle \theta \left[ \int _{0}^{l}\beta (x)T^{(\lambda )}(x,\bar{x})\mathrm{d}x -\sum _{j=0}^{N}w_{N,j}\beta (x_{N,j})T^{(\lambda )}(x_{N,j},\bar{x})\right] \nonumber \\&\quad \displaystyle +\theta \sum _{j=0}^{N}w_{N,j}\beta (x_{N,j}) \left[ T^{(\lambda )}(x_{N,j},\bar{x})-T^{(\lambda )}_{N}(x_{N,j},\bar{x})\right] . \end{aligned}$$

As it follows from (63) and (70), the first and the third addends in the right-hand side above depend on the collocation error defined as

74 $$\begin{aligned} e_{N}:=p_{N}-\phi \end{aligned}$$

for $p_{N}$ the collocation polynomial satisfying (69) and $\phi$ the solution of (59). Indeed

75 $$\begin{aligned} T^{(\lambda )}_{N}(x,\bar{x})-T^{(\lambda )}(x,\bar{x}) =\frac{e_{N}(x)}{\alpha },\quad x\in [0,l], \end{aligned}$$

holds for any $\alpha \in {\mathbb {R}}\setminus \{0\}$, as assumed in (59). Prior to analyze this contribution to the error on the characteristic function, we first recall a general (and known) result on the quadrature error based on (23), holding under (A3). On the one hand the latter clearly serves to bound the second addend above. On the other hand it affects also the analysis of the collocation error.

##### Lemma 2

(**see Theorem 4.1 in** [38]) Let $E_{N}(f)$ be the best uniform approximation error of a continuous function $f:[0,l]\rightarrow {\mathbb {R}}$ in $\varPi _{N}$ (the set of algebraic polynomials of degree $\le N$ in [0, *l*]). Then, under (A3),

76 $$\begin{aligned} \left| \int _{0}^{l}f(x)\mathrm{d}x-\sum _{j=0}^{N}w_{N,j}f(x_{N,j})\right| \le 2lE_{N}(f) \end{aligned}$$

and $E_{N}(f)$ vanishes as $N\rightarrow \infty$.

Going back to the collocation error (74), subtracting (66) from (72) yields the functional equation in *X*

77 $$\begin{aligned} e_{N}=V{\mathcal {L}}_{N-1}A^{(\lambda )}_{N}e_{N}+Vr_{N} \end{aligned}$$

with

78 $$\begin{aligned} r_{N}:=\left( {\mathcal {L}}_{N-1}A^{(\lambda )}_{N}-A^{(\lambda )}\right) \phi \end{aligned}$$

for $\phi$ the solution of (59).

##### Proposition 6

Under (HB4) and (A3), there exists $N^{*}\in {\mathbb {N}}$ such that for all the integers $N\ge N^{*}$ (77) has a unique solution $e_{N}$ and

79 $$\begin{aligned} \Vert e_{N}\Vert _{\infty }\le 2\left\| \left( I_{X}-A^{(\lambda )} V\right) ^{-1}\right\| _{X\leftarrow X}\Vert r_{N}\Vert _{X}. \end{aligned}$$

##### Proof

The proof is similar to that of Proposition 3, so that together with (77) we consider also the functional equation in *X*

80 $$\begin{aligned} \tilde{e}_{N}={\mathcal {L}}_{N-1}A^{(\lambda )}_{N}V\tilde{e}_{N}+r_{N}, \end{aligned}$$

noting that their solutions are in one-to-one correspondence through $e_{N}=V\tilde{e}_{N}$ and $\tilde{e}_{N}={\mathcal {L}}_{N-1} A^{(\lambda )}_{N}e_{N}+r_{N}$. We prove then that (80) admits a unique solution for *N* large enough by showing through the Banach’s perturbation lemma (see, e.g., Theorem 10.1 in [24]) that $I_{X}-{\mathcal {L}}_{N-1}A^{(\lambda )}_{N}V$ is invertible with (uniformly) bounded inverse. To this aim we need to prove that (a) $I_{X}-A^{(\lambda )}V$ is invertible with bounded inverse and that (b) $\Vert ({\mathcal {L}}_{N-1}A^{(\lambda )}_{N} -A^{(\lambda )})V\Vert _{X\leftarrow X}$ vanishes as $N\rightarrow \infty$. As for (a) let us show that, given any $\psi \in X$, $(I_{X}-A^{(\lambda )}V)\phi =\psi$ has a unique solution. Indeed, the latter translates into $\xi '=A^{(\lambda )}\xi +\psi$ for $\xi :=V\phi$, which reads

$$\begin{aligned} \xi =g+L\xi \end{aligned}$$

for *L* in (62) and

$$\begin{aligned} g(x):=\int _{\bar{x}}^{x}T(x,y)\xi (y)\mathrm{d}y,\quad x\in [0,l]. \end{aligned}$$

Then the same arguments used to prove Proposition 5 applies and (a) is proved. As for (b) let us write

$$\begin{aligned} \left( {\mathcal {L}}_{N-1}A^{(\lambda )}_{N}-A^{(\lambda )}\right) V =({\mathcal {L}}_{N-1}-I_{X})A^{(\lambda )}_{N}V+\left( A^{(\lambda )}_{N} -A^{(\lambda )}\right) V \end{aligned}$$

and observe that *V*(*X*) is a subset of the space $C\subset X$ of continuous functions. The same holds for $A^{(\lambda )}V(X)$ under (HB4). This guarantees that the second addend above vanishes as $N\rightarrow \infty$ thanks to Lemma 2. Also the functions in $A^{(\lambda )}_{N}V(X)$ are continuous. Moreover, they are uniformly bounded under (A3) since the corresponding quadrature is convergent and hence $\sum _{j=0}^{N}w_{N,j}$ is uniformly bounded. Then the first addend vanishes as $N\rightarrow \infty$ since $\Vert {\mathcal {L}}_{N-1}-I_{X}\Vert _{X\leftarrow C}$ vanishes thanks to Corollary of Theorem Ia in [14]. Eventually, $e_{N}=V\tilde{e}_{N}$ gives $\Vert e_{N}\Vert _{\infty }\le \Vert \tilde{e}_{N}\Vert _{X}$ and the thesis follows similarly as in the proof of Proposition 3. $\square$

Now, in view of (79) and similarly to Sect. 4.1.3, we evaluate the remainder $r_{N}$ as defined in (78), a bound of which depends on the smoothness of the map $x\mapsto A^{(\lambda )}(x)$ (and thus on that of $\eta$ and *K*). Let us recall that this bound will be used below to estimate (73) from above.

##### Proposition 7

Under (HB5) and (A3), there exists $N^{*}\in {\mathbb {N}}$ such that for all the integers $N\ge N^{*}$

$$\begin{aligned} \Vert r_{N}\Vert _{X}\le \varepsilon _{N}|\alpha | \end{aligned}$$

with

$$\begin{aligned} \varepsilon _{N}={\left\{ \begin{array}{ll} O(N^{-s}\log N) &{}\text {under (HB5.1)} \\ O(N^{-r}\log N) &{}\text {for every integer}\ r\ge 1\ \text {under (HB5.2)}\\ O(k^{-N}\log N) &{}\text {for some constant}\ k>1\ \text {under (HB5.3).} \end{array}\right. } \end{aligned}$$

##### Proof

From (78) we have

$$\begin{aligned} \left\| \left( {\mathcal {L}}_{N-1}A^{(\lambda )}_{N} -A^{(\lambda )}\right) \phi \right\| _{X}\le l \left\| ({\mathcal {L}}_{N-1}-I_{X})A^{(\lambda )}_{N} \phi \right\| _{\infty }+l\left\| \left( A^{(\lambda )}_{N} -A^{(\lambda )}\right) \phi \right\| _{\infty }. \end{aligned}$$

The first addend above is bounded according to the same arguments used in the proof of Proposition 4 by recalling, in addition, the uniform boundedness of the functions in the range of $A^{(\lambda )}_{N}$ as discussed in the proof of Proposition 6. As for the second addend, one order more is obtained according to Lemma 2 since the Lebesgue constant is not involved in the bound (76). The final bound follows by considering that $\phi$ depends linearly on $\alpha$ through (63). $\square$

##### Proposition 8

Under (HB5), (HB6) and (A3), there exists $N^{*}\in {\mathbb {N}}$ such that for all the integers $N\ge N^{*}$

81 $$\begin{aligned} |g_{N}(\lambda )-g(\lambda )|\le \varepsilon _{N} \end{aligned}$$

with

$$\begin{aligned} \varepsilon _{N}={\left\{ \begin{array}{ll} O(N^{-s}\log N) &{}\text {under (HB5.1) and (HB6.1)} \\ O(N^{-r}\log N) &{}\text {for every integer}\ r\ge 1\ \text {under (HB5.2) and (HB6.2)}\\ O(k^{-N}\log N) &{}\text {for some constant}\ k>1\ \text {under (HB5.3) and (HB6.3).} \end{array}\right. } \end{aligned}$$

##### Proof

From (73) we have

$$\begin{aligned} |g_{N}(\lambda )-g(\lambda )|&\le \displaystyle \left| T^{(\lambda )}_{N} (0,\bar{x})-T^{(\lambda )}(0,\bar{x})\right| \\&\quad +\displaystyle \left| \int _{0}^{l}\beta (x)T^{(\lambda )} (x,\bar{x})\mathrm{d}x-\sum _{j=0}^{N}w_{N,j}\beta (x_{N,j})T^{(\lambda )} (x_{N,j},\bar{x})\right| \\&\quad \displaystyle +l\Vert \beta \Vert _{\infty }\left\| T^{(\lambda )} (\cdot ,\bar{x})-T^{(\lambda )}_{N}(\cdot ,\bar{x})\right\| _{\infty }. \end{aligned}$$

The thesis follows by applying Propositions 6 and 7 through (75) as far as the first and the third addends in the right-hand side above are concerned. Additionally, Lemma 2 provides the similar bound for the second addend by taking into account also the hypothesis (HB6) on $\beta$ (but with one order less for the same reasons as in the proof of Proposition 7). $\square$

We conclude with the main result for the class of epidemic models (model B), direct consequence of Theorem 1 and Proposition 8.

##### Theorem 3

Let $\lambda ^{*}$ be a zero of *g* in (65) with algebraic multiplicity *m* and $r>0$ be such that $\lambda ^{*}$ is the only zero of *g* in $\overline{B}(\lambda ^{*},r)$. Then, under (HB5), (HB6) and (A3), there exists $N^{*}\in {\mathbb {N}}$ such that, for all the integers $N\ge N^{*}$, $g_{N}$ in (71) has exactly *m* zeros $\lambda _{N,1},\dots ,\lambda _{N,m}$ (counted with multiplicities) in $\overline{B}(\lambda ^{*},r)$ and

$$\begin{aligned} \max _{i=1,\dots ,m}|\lambda ^{*}-\lambda _{N,i}|\le \rho (N) \end{aligned}$$

with

$$\begin{aligned} \rho _{N}={\left\{ \begin{array}{ll} O((N^{-s}\log N)^{1/m}) &{}\text {under (HA5.1) and (HB6.1)} \\ O((N^{-r}\log N)^{1/m}) &{}\text {for every integer}\ r\ge 1\ \text {under (HA5.2)}\\ &{}\text {and (HB6.2)}\\ O((k^{-N}\log N)^{1/m}) &{}\text {for some constant}\ k>1\ \text {under (HA5.3)}\\ &{}\text {and (HB6.3).} \end{array}\right. } \end{aligned}$$

## Numerical Tests

We present a series of experiments with the principal aim at giving practical confirmation of the convergence results of Sect. 4. In particular, we focus also on the effect of the (lack of) smoothness of the model coefficients as well as of the (lack of) compactness of the relevant NGO.

To this aim, we investigate the behavior of both the error $|R_{0,N}-R_{0}|$ between the approximated and the exact basic reproduction numbers and the error $\Vert \phi _{N}-\phi \Vert _{\infty ,M}$ between the relevant exact generalized eigenfunction and its collocation approximation. The latter error is measured as the maximum absolute value of the difference of the two functions on a mesh of *M* equidistant points in [0, *l*]. Let us remark that $\phi _{N}$ is reconstructed from the computed generalized eigenvector $\varPhi$ associated to the dominant generalized eigenvalue of (25) through barycentric interpolation [4]. Above, with *exact* we mean either the theoretical value (or expression) when explicitly available, or a reference counterpart $R_{0,\bar{N}}$ (or $\phi _{\bar{N}}$) computed with a given large $\bar{N}$ otherwise. In all the following tests we use $\bar{N}=M=1\,000$.

In Sect. 5.1 below we list all the specific choices of the concerned models by giving the defining rates and coefficients. We also give some of their key analytical features when available, e.g., exact values for $R_{0}$ and possibly for the relevant generalized eigenfunction, respectively $\lambda$ and $\phi$ in (5)^9^. Results and relevant comments are then presented in Sect. 5.2.

Note that most of the following choices differ from those presented in [5] in order to provide the reader with a larger benchmark. Moreover, and again differently from [5], we restrain to give biological interpretations as this work focuses on the numerical analysis. Finally, all the experiments are run on a MacBook Pro 2.3 GHz Intel Core i7 with 16 GB memory, through codes written in Matlab R2019a (codes freely available at http://cdlab.uniud.it/software). In particular, let us remark that the discrete generalized eigenvalue problem (25) is solved by using eig(B/M) rather than eig(B,M), for related comments see Section 4.1.2 in [5].

### Model Choices

For all the instances of model A listed next we set $l=1$ and, for $x\in [0,l]$,

$$\begin{aligned} c(x):=l^{2}-x^{2},\qquad D(x):=\tilde{D} \cdot \left[ 2+\sin (f\pi x)\right] \ge \tilde{D}, \end{aligned}$$

with $f=1$ if not differently specified. All the other ingredients are described below.

1. 10 Let $\tilde{D}=2$, $\beta (x):=\tilde{\beta }=3$ and $\mu (x):=\tilde{\mu }=1$. With these choices the NGO is clearly compact (recall Proposition 1) and thus $R_{0}$ is a generalized eigenvalue. Some calculations that we omit allow to recover
  $$\begin{aligned} R_{0}=\frac{2\tilde{\beta }}{\tilde{\beta }+\tilde{\mu }}=1.5, \end{aligned}$$
  with corresponding generalized eigenfunction
  $$\begin{aligned} \phi (x)=e^{\int _{0}^{x}\frac{c(y)}{D(y)}\mathrm{d}y},\quad x\in [0,l], \end{aligned}$$
  normalized as $$\phi (0)=1$$.
2. For $$x\in [0,l]$$, let
  $$\begin{aligned} \mu (x):=\tilde{\mu }\cdot \frac{x}{l},\qquad \beta (x) :=\tilde{\beta }\cdot \left[ \frac{27}{2l^{3}}x^{2}(l-x)+1\right] \end{aligned}$$
  with $\tilde{\mu }=1$ and $\tilde{\beta }=3$. With respect to Proposition 1 we consider either
  (T2.1A) a compact case: $$\tilde{D}=2$$;
  (T2.2A) a “almost non-compact” case: $\tilde{D}=10^{-6}$;
  (T2.3A) the non-compact case: $\tilde{D}=0$.^11^
  Independently of the choices of $\tilde{D}$, both $R_{0}$ and the corresponding eigenfunction are unknown.
3. Let $\tilde{D}=2$, $\mu$ be the same as in case (T2A) and
  $$\begin{aligned} \beta (x):={\left\{ \begin{array}{ll} \tilde{\beta },&{} x\in [0, l_{0}),\\ 2\tilde{\beta },&{} x\in [l_{0}, l], \end{array}\right. } \end{aligned}$$
  with $\tilde{\beta }=3$ and $l_{0}=l/2$. The NGO is compact (recall Proposition 1), but $R_{0}$ and the corresponding generalized eigenfunction are unknown.

For all the instances of model B we set again $l=1$ and the rest is defined below.

1. For $\tilde{k}$ and $\alpha$ both positive let
  $$\begin{aligned} K(x,y):=\tilde{k} x^{2}(l-x)^{2}\cdot \frac{\varPi _{0}(y)}{\int _{0}^{l} \varPi _{0}(z)\mathrm{d}z},\quad x,y\in [0,l], \end{aligned}$$
  with $\varPi _{0}(x):=\left( 1-x/l\right) ^{\alpha }$. Let, moreover,
  $$\begin{aligned} \eta (x):=\frac{1}{l-x},\qquad \beta (x):= \frac{\alpha +1}{l} \left( \frac{l-x}{l}\right) ^{\alpha } \end{aligned}$$
  for $x\in [0,l]$^12^. With these choices the NGO is compact (recall Proposition 2). Since *K* above is the product of functions in each of the variables *x* and *y*, the NGO becomes a rank one operator and we can explicitly compute
  $$\begin{aligned} R_{0}=\frac{2\tilde{k}(\alpha +1)l^{5}}{(\alpha +2-\theta (\alpha +1)) (\alpha +4)(\alpha +5)(\alpha +6)}. \end{aligned}$$
  We fix $R_{0}=2.5$ for different values of $\alpha$ and $\theta$ as follows:
  (T1.1B) $$\alpha =1/4$$, $$\theta =2/5$$ and $$\tilde{k}= 62\,475/256$$;
  (T1.2B) $$\alpha =1/4$$, $$\theta =0$$ and $$\tilde{k}= 80\,325/256$$;
  (T1.3B) $$\alpha =1$$, $$\theta =2/5$$ and $$\tilde{k} =1\,155/4$$;
  (T1.4B) $$\alpha =1$$, $$\theta =0$$ and $$\tilde{k}= 1\,575/4$$.
  Finally, the relevant generalized eigenfunction is
  $$\begin{aligned} \phi (x)=(1-x/l)\left( C +l \int _{0}^{x}\frac{(l-y)y^{2}}{(1-y/l)}\mathrm{d}y\right) ,\quad x\in [0,l], \end{aligned}$$
  with
  $$\begin{aligned} C:=\frac{2\theta (\alpha +1)l^{5}}{(\alpha +2-\theta (\alpha +1)) (\alpha +4)(\alpha +5)(\alpha +6)} = \frac{\theta }{\tilde{k}} R_{0}, \end{aligned}$$
  which, for the choices above, turns out to be a polynomial of degree 5, viz.
  82 $$\begin{aligned} \phi (x)=(l-x)\left( \frac{C}{l}+\frac{x^{3}(4l-3x)}{12}\right) ,\quad x\in [0,l], \end{aligned}$$
  normalized as $$\phi (l/2)=C/2+5l^{5}/384$$.
2. For $l_{0}=0.1l$ and $\alpha =0.1$ let
  $$\begin{aligned} K(x,y):=\tilde{k} e^{-f(x,y)}\cdot \frac{\varPi _{0}(y)}{\int _{0}^{l}\varPi _{0}(z)\mathrm{d}z},\quad x,y\in [0,l], \end{aligned}$$
  with either
  (T2.1B) $$f(x,y):=|x-y|/l_{0}$$, $$\theta =0$$ and $$\tilde{k}=141$$;
  (T2.2B) $$f(x,y):=|x-y|/l_{0}$$, $$\theta =2/5$$ and $$\tilde{k}=124$$;
  (T2.3B) $$f(x,y):=(x-y)^{2}/l_{0}^{2}$$, $$\theta =0$$ and $$\tilde{k}=189$$;
  (T2.4B) $$f(x,y):=(x-y)^{2}/l_{0}^{2}$$, $$\theta =2/5$$ and $$\tilde{k}=137$$;
  and $\varPi _{0}(x):=e^{-\alpha x(l-x)}$. Some calculations that we omit give
  $$\begin{aligned} \int _{0}^{l}\varPi _{0}(x)\mathrm{d}x=l\left( 1-\alpha e^{\alpha }\int _{\alpha }^{+\infty } \frac{e^{-x}}{x}\mathrm{d}x\right) . \end{aligned}$$
  Let also
  $$\begin{aligned} \eta (x):= \tilde{\eta }=9,\qquad \beta (x):=\frac{b(x)\varPi _{0}(x)}{\int _{0}^{l} b(y)\varPi _{0}(y)\mathrm{d}y} \end{aligned}$$
  for $x\in [0,l]$ and $b(x):=(x/l)^{2}e^{-6x/l}$. For these choices the NGO is compact (recall Proposition 2), but both $R_{0}$ and the corresponding eigenfunction are unknown.

### Results

**Fig. 1:**
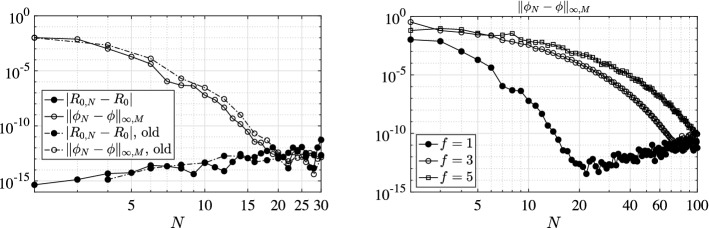
Left—(T1A) spectral convergence; right—(T1A) effect of varying *f* in *D*. See text for more details

**Fig. 2:**
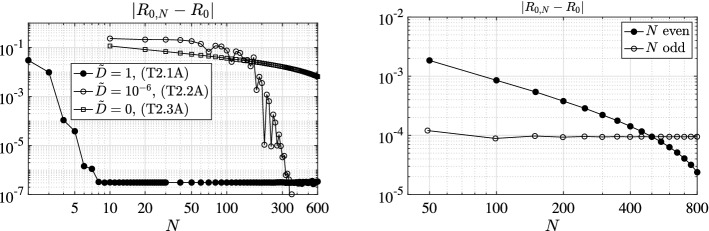
Left—(T2A) error for increasing *N* on $R_{0}\simeq 1.853050859715361$ for (T2.1A) (bullets), $R_{0}\simeq 1.504307446573413$ for (T2.2A) (circles), $R_{0}\simeq 1.621272061691218$ for (T2.3A) (squares); right—(T3A) error for increasing *N* on $R_{0}\simeq 1.798169690353490$. See text for more details

**Fig. 3:**
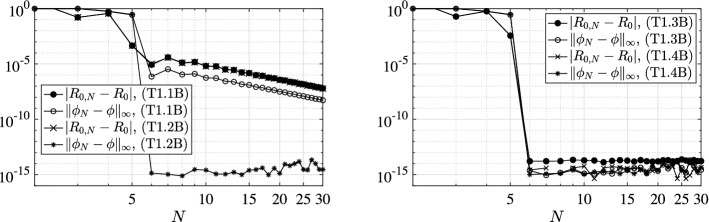
Left—convergence for (T1.1B) and (T1.2B), i.e., $\alpha =1/4$; right—convergence for (T1.3B) and (T1.4B), i.e., $\alpha =1$. See text for more details

**Fig. 4:**
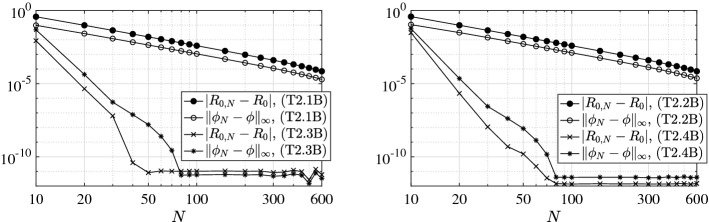
Left—convergence to $R_{0,\bar{N}}\simeq 2.499486715274162$ for (T2.1B) and to $R_{0,\bar{N}}\simeq 2.506858993699479$ for (T2.3B), i.e., $\theta =0$; right—convergence to $R_{0,\bar{N}}\simeq 2.504893610018024$ for (T2.2B) and to $R_{0,\bar{N}}\simeq 2.501711815944655$ for (T2.4B), i.e., $\theta =2/5$. See text for more details

(T1A) Fig. 1 (left) shows a spectrally accurate behavior – the error decays faster than $O(N^{-k})$ for any natural *k*, [37] – for the approximation of the generalized eigenfunction $\phi$ (solid line with circles), confirming Theorem 2 under (HA5.3). The error on $R_{0}$ (solid line with bullets) is instead around machine precision already at low values of *N* (with a mild algorithmic instability appearing at larger values). Indeed, with the choice of constant vital rates it is not difficult to show that (6) can be reduced to a scalar ODE, so that the associated eigenvalue problem has dimension 1, and it is in fact approximated very well even with low values of *N*. The same panel reports also the results obtained with the discretization originally presented in [5] (dashed-dotted lines): as anticipated in Remark 1 they are practically indistinguishable. In Fig. 1 (right) we instead investigate the case of varying $f=1,3,5$ in *D*. While we omit to plot the convergence to the eigenvalue since unaffected (being the relevant problem of dimension 1 as explained above), it can be seen that the convergence to the eigenfunction is slowed down as *f* increases, still being the error spectrally accurate. This is in perfect agreement with the convergence analysis: the convergence is spectral since *D* is smooth (indeed real analytic), yet the error constant is proportional (also) to the growth of the derivatives of *D*, and hence to *f*. In this respect see the proof of Proposition 4 and the dependence on the derivatives of $A^{(\lambda )}\eta$ for $A^{(\lambda )}$ in (30).

(T2A) The results reported in Fig. 2 (left) about the error with respect to the reference value $R_{0,\bar{N}}$ show spectral accuracy for (T2.1A), where compactness of the NGO is ensured according to Proposition 1. Theoretically, also (T2.2A) guarantees compactness, but it is clearly visible from the plot that much larger values of *N* are necessary to start appreciating the spectral accuracy. Indeed, given (30) and its role in the convergence analysis, it is reasonable that the value of $\tilde{D}$ affects the error constants, causing their increase as $\tilde{D}\rightarrow 0$, still being the problem compact as far as $\tilde{D}>0$ as assumed in HA3. When we deal instead with (T2.3A), where the absence of diffusion causes the loss of compactness, convergence still occurs, even though at a fixed rate (seemingly linear). The fact is somehow surprising (and certainly merits future investigation), given that in absence of compactness (5) may even become meaningless and we are thus using a finite-dimensional eigenvalue problem to approximate components of the spectrum possibly other than the point one. See also [5, Figure 3] for a similar example.

(T3A) As expected from Theorem 2, convergence is not attained as $\beta$ is not even continuous, Fig. 2 (right). This is true for *N* odd (solid line with circles), even though the error is anyway small. Yet unsurprisingly, convergence is scored with *N* even (solid line with bullets) since, under (A3), the discontinuity point *l*/2 is always included in the discretization mesh. Note, however, that the rate appears to be only linear. In this respect, let us remark that a piecewise collocation is the only reasonable remedy to the issue, and the authors reserve to investigate this generalization in a future work.

(T1B) Starting from (T1.1B), Fig. 3 (left, solid lines) shows convergence to both $R_{0}$ and the relevant eigenfunction, but with trends different from what experimented so far. Concerning the approximation of $R_{0}$, according to Theorem 3 the lack of smoothness of $\beta$ for $\alpha =1/4<1$ (being rational and blowing up at $x=l$) prevents the method to perform the standard spectral accuracy, and convergence of fixed order (seemingly 4) occurs. The same trend is observed also concerning the approximation of the eigenfunction. Indeed, recall from (82) that the latter is a polynomial of degree 5, which justifies the visible drop of the error occurring with $N=6$. Yet $\phi$ depends on the constant *C*, and hence on $R_{0}$, causing the convergence of fixed order as explained above. Passing to (T1.2B), Fig. 3 (left, dashed-dotted lines), we see that the same behavior is observed concerning the convergence to $R_{0}$. Instead, with respect to the eigenfunction, a sudden drop of the error to machine precision occurs with $N=6$ in perfect accordance with Proposition 7. Here there is no effect of the approximation of $R_{0}$ since the constant *C* in (82) vanishes being $\theta =0$. As far as (T1.3B) and (T1.4B) are concerned, Fig. 3 (right), the just mentioned sudden drop of the error for $N=6$ occurs for both the approximation of $R_{0}$ and the relevant eigenfunction, independently of the value of $\theta$. Indeed, these cases share the common value $\alpha =1$, for which $\beta$ is linear and hence enough smoothness is granted.

(T2B) According to Theorem 3, convergence is not guaranteed for (T2.1B) given the lack of smoothness of *K*, yet not ruled out being the result a sufficient condition. In fact the method is still able to converge, seemingly with order 2 (both to $R_{0}$ and to the eigenfunction), Fig. 4 (left). An explanation of this positive behavior relies on the hyphotesis (HB5) on *K* in Theorem 3, which is about the map $x\mapsto K(x,y)$ for almost all *y*. In this respect it happens that for every mesh point *y*, the discontinuity which arises only at $x=y$ is included in the relevant mesh, as the latter is necessarily the same for both directions. For the sake of comparison, Fig. 4 (left) reports also the results about the smooth kernel of (T2.3B), the same considered in case (B3) of [5]. For the latter, smoothness guarantees the expected spectrally accurate behavior. Both tests concern $\theta =0$. The case $\theta =2/5$ of (T2.2B) and (T2.4B) is illustrated in Fig. 4 (right), where no difference arises with respect to the behavior above described.

## Concluding Remarks

In this paper we contribute a full analysis of theoretical (compactness) and numerical (error and convergence) aspects of the collocation approach proposed in [5] to compute the basic reproduction number of structured population dynamics. On the one hand, we prove under mild regularity assumptions of the models coefficients that the concerned operators are compact, so that the problem can be properly recast as an eigenvalue problem thus allowing for discretization (Sect. 3). On the other hand, we prove through detailed and rigorous error and convergence analyses that the method indeed performs the expected spectral accuracy as thoroughly experimented in [5] (Theorems 2 and 3).

Let us recall that $R_{0}$ is a key quantity in addressing the evolution of structured populations describing realistic phenomena in both ecology and epidemiology. As for the latter, for instance, the epidemic character of diseases like COVID-19 highly depends on the age, location and other heterogeneity of the host population. Therefore, computing $R_0$ for epidemic models with multiple structuring traits is quite important from a practical viewpoint. Extension of the proposed approach in this direction resorting to multivariate collocation is, in fact, among the plans of the authors (and colleagues).

Also several other directions are already under consideration, a brief summary of which follows.

The investigated approach heavily relies on the compactness of the NGO. Yet some of the numerical experiments clearly show that reasonable approximations to $R_{0}$ can be obtained even if the latter is not (necessarily) an eigenvalue. The relevant analysis demands for non-standard theoretical and numerical tools as one should aim at approximating parts of the spectrum other than the point one.

In the current work we assume the birth operator to be bounded, see Note 2. Though being a common assumption, there are models relying on unbounded *B* (see, e.g., [1]) yet with bounded NGO. Thus an extension in this respect is worth a try.

From the numerical standpoint, other valuable (yet perhaps more technical) extensions on which the authors plan to work include the multivariate case $X^{n}$ mentioned in Note 1, as well as the piecewise extension as arisen in commenting (T3A).

Finally, a last direction with much relevance in applications is towards models with time-periodic or even time-heterogeneous coefficients [20, 22], for which a numerical treatment completely lacks.

## A Auxiliary Results

Propositions 9 and 10 below are used in the proof of Proposition 5 in Sect. 4.2 and concern the integral operator *L* defined in (62). Prior to prove them note that the function *T* introduced in (20) satisfies

83 $$\begin{aligned} T(x,y)=T(x,z)T(z,y) \end{aligned}$$

for every $x,y,z\in [0,l]$ and

84 $$\begin{aligned} T(x,0)^{-1}=T(0,x) \end{aligned}$$

for every $$x\in [0,l]$$.

### Proposition 9

Under (HB1) and (HB2) *L* is compact.

### Proof

The proof follows the same lines of that of Proposition 2 (and hence that of Proposition 1), once observed that *L* in (62) is the same integral operator appearing in (22), but for the lower integration extremum $\bar{x}$ replacing 0 and $B\varphi /\lambda$ replacing $\psi$. As for the latter, note that *B* is bounded under (HB2). $\square$

### Proposition 10

Let $\bar{x}\in [0,l]$ in (59). Under (HB1) $I_{X}-L$ is injective if and only if $\bar{x}\in (0,l]$.

### Proof

We first prove that $\bar{x}=0$ implies $I_{X}-L$ not injective. To this aim we look for a nontrivial $\varphi \in X$ satisfying

$$\begin{aligned} \varphi (x)=\int _{0}^{x}T(x,y)\frac{1}{\lambda }(B\varphi )(y)\mathrm{d}y,\quad x\in [0,l]. \end{aligned}$$

Equivalently, $\varphi$ solves the IVP

$$\begin{aligned} \left\{ \begin{array}{l} \varphi '(x)=\displaystyle -\eta (x)\varphi (x) +\frac{1}{\lambda }(B\varphi )(x),\quad x\in [0,l],\\ \varphi (0)=0. \end{array}\right. \end{aligned}$$

Then it is clear that such a $\varphi$ exists since it is an eigenfunction of the generalized eigenvalue problem (5) in the case $\theta =0$.

Next we assume $\bar{x}\in (0,l]$ and prove that $I_{X}-L$ is injective. We proceed by contradiction, thus letting $\varphi \in X\setminus \{0\}$ be such that

85 $$\begin{aligned} \varphi (x)=\int _{\bar{x}}^{x}T(x,y)\frac{1}{\lambda } (B\varphi )(y)\mathrm{d}y,\quad x\in [0,l]. \end{aligned}$$

Note that $\varphi (\bar{x})=0$ together with

86 $$\begin{aligned} \varphi (x)&=\displaystyle \int _{0}^{x}T(x,y)\frac{1}{\lambda } (B\varphi )(y)\mathrm{d}y-\int _{0}^{\bar{x}}T(x,y)\frac{1}{\lambda }(B\varphi )(y)\mathrm{d}y\nonumber \\&=\displaystyle \frac{1}{\lambda }(M^{-1}B\varphi )(x)-T(x,0)H\left( \frac{1}{\lambda } B\varphi ;\theta \right) \nonumber \\&\quad \displaystyle -T(x,\bar{x})\left[ \frac{1}{\lambda } (M^{-1}B\varphi )(\bar{x})-T(\bar{x},0)H\left( \frac{1}{\lambda } B\varphi ;\theta \right) \right] \nonumber \\&=\displaystyle \frac{1}{\lambda }(M^{-1}B\varphi )(x)-T(x,\bar{x}) \frac{1}{\lambda }(M^{-1}B\varphi )(\bar{x}) \end{aligned}$$

as it follows from (22) in the proof of Proposition 2. Observe that (85) implies also

$$\begin{aligned} \varphi (0)=\int _{\bar{x}}^{0}T(0,y)\frac{1}{\lambda }(B\varphi )(y)\mathrm{d}y. \end{aligned}$$

Assume $\varphi (0)>0$. Since $\varphi$ is continuous, there exists some $\hat{x}\in (0,\bar{x}]$ such that $\varphi (x)>0$ for every $x\in [0,\hat{x})$ and $\varphi (\hat{x})=0$. Assume also $\hat{x}<\bar{x}$. Then the absurd

$$\begin{aligned} 0<\varphi (0)&=\displaystyle \int _{\hat{x}}^{0}T(0,y) \frac{1}{\lambda }(B\varphi )(y)\mathrm{d}y+\int _{\bar{x}}^{\hat{x}}T(0,y) \frac{1}{\lambda }(B\varphi )(y)\mathrm{d}y\\&=\displaystyle \int _{\hat{x}}^{0}T(0,y)\frac{1}{\lambda }(B\varphi ) (y)\mathrm{d}y+T(0,\hat{x})\varphi (\hat{x})\\&=\displaystyle \int _{\hat{x}}^{0}T(0,y)\frac{1}{\lambda }(B\varphi )(y)\mathrm{d}y\\&<0 \end{aligned}$$

since $\lambda >0$ (as spectra radius of a compact positive operator), *T* is positive and *B* has non-negative kernel under (HB1). Let therefore $\hat{x}=\bar{x}$, but the absurd follows again as

$$\begin{aligned} 0<\varphi (0)=\int _{\bar{x}}^{0}T(0,y)\frac{1}{\lambda }(B\varphi )(y)\mathrm{d}y<0 \end{aligned}$$

by the same reasoning. As the case $\varphi (0)<0$ is equally ruled out, we conclude that $\varphi (0)=0$.

Now recall that the definition of *L* as well as its properties are independent of the parameter $\theta$ characterizing ${\mathcal {D}}(M)$, so that we are free to choose any value for the latter. For the sake of simplicity let us set then $\theta =0$. Accordingly, *H* in (21) vanishes and hence

$$\begin{aligned} \varphi (0)=\int _{\bar{x}}^{0}T(0,y)\frac{1}{\lambda }(B\varphi )(y) \mathrm{d}y=-T(0,\bar{x})\frac{1}{\lambda }(M^{-1}B\varphi )(\bar{x}) \end{aligned}$$

follows from (22) by applying (83). Then, by applying (84), $(M^{-1}B\varphi )(\bar{x})=-\lambda T(\bar{x},0)\varphi (0)=0$ and hence (86) becomes

$$\begin{aligned} \varphi (x)=\frac{1}{\lambda }(M^{-1}B\varphi )(x),\quad x\in [0,l]. \end{aligned}$$

Moreover, $\theta =0$ and $\varphi (0)=0$ imply $\varphi \in {\mathcal {D}}(M)$, so that the latter equation is equivalent to

$$\begin{aligned} B\varphi =\lambda M\varphi . \end{aligned}$$

Since $\lambda$ is the spectral radius of the positive compact operator $BM^{-1}$, the Krein-Rutman theorem [23] ensures that there exists a positive eigenfunction $\xi$ of $BM^{-1}$ and hence it easily follows from (22) that $\varphi =M^{-1}\xi$ is non-negative and it vanishes only at $x=0$, which contradicts the existence of $\bar{x}\in (0,l]$ such that $\varphi (\bar{x})=0$ according to (85). Therefore there is no $\varphi \in X\setminus \{0\}$ such that $\varphi =L\varphi$ and thus $I_{X}-L$ is injective. $\square$

## Footnotes

1 The proposed numerical approach can be applied also when $u\in X^{n}$ for some positive integer *n*, resorting to a straightforward block-wise structure. This is, e.g., the case of an epidemics with multi-type sets of infective individuals in a broad sense (e.g., exposed, asymptomatic, mild/moderate/severe infected, etc.).

2 A possible extension of the present approach to the case of unbounded *B* yet with bounded $BM^{-1}$ is currently under consideration by the authors.

3 Note that the Krein-Rutman theorem for compact and positive operators does not tell anything about the multiplicity of the spectral radius as eigenvalue. The version for strongly positive operators (Proposition 10.9 in Section 10.2 of [21]) guarantees the latter to be simple, but it requires the lattice *X* to have non-empty interior, which is not the case for the choice of *X* we make in the sequel, i.e., in $L^{1}$. For this choice we have instead a total cone, and Propositions 10.10 and 10.11 in Section 10.2 of [21] apply if the NGO is semi-nonsupporting or nonsupporting.

4 Actually, $u(\cdot ,t)$ is a weighted density obtained by removing the age-specific mortality rate, see Model B in [5].

5 We recall that here birth is meant as infection.

6 We recall that here mortality is meant as recovery and/or any other stage transition.

7 Here $a_{N;i,j}$ denotes the (*i*, *j*)-th entry of a matrix $A_{N}\in {\mathbb {R}}^{(N+1)\times (N+1)}$, $i,j=0,1,\ldots ,N$. This notation is also extended later on according to Matlab’s *colon* notation: for instance $a_{N;i,0:N}$ and $a_{N;0:N,j}$ indicate, respectively, the *i*-th row and the *j*-th column of $A_{N}$.

8 Usually these quantities are provided with reference to the normalized interval $[-1,1]$.

9 Hereafter, for brevity, we omit the adjective *generalized* given as granted the reference to (5).

10 The label (T$\#$A) stands for test number $\#$ on model A (the same is used for model B below).

11 In this case $R_{0}$ can be computed analytically and it turns out not to be an eigenvalue (see a similar computation in Section 3.1 of [3]).

12 For this specific choice of $\eta$, and for the sake of pragmatism, with reference to (A3) here we exclude the last node $x_{N,N}=l$ from the computations to avoid overflow. However, the issue can be avoided properly by using meshes that exclude this extremum by definition, e.g., Chebyshev zeros.
